# Anti-Inflammatory, Antiallergic, and COVID-19 Main Protease (M^pro^) Inhibitory Activities of Butenolides from a Marine-Derived Fungus *Aspergillus terreus*

**DOI:** 10.3390/molecules26113354

**Published:** 2021-06-02

**Authors:** Ibrahim Seyda Uras, Sherif S. Ebada, Michal Korinek, Amgad Albohy, Basma S. Abdulrazik, Yi-Hsuan Wang, Bing-Hung Chen, Jim-Tong Horng, Wenhan Lin, Tsong-Long Hwang, Belma Konuklugil

**Affiliations:** 1 Department of Pharmacognosy, Faculty of Pharmacy, Ankara University, Ankara 06560, Turkey; isuras@ankara.edu.tr; 2 Department of Pharmacognosy, Faculty of Pharmacy, Agri Ibrahim Cecen University, Agri 04100, Turkey; 3 Department of Pharmacognosy, Faculty of Pharmacy, Ain Shams University, Abbasia, Cairo 11566, Egypt; 4 Department of Pharmacognosy, Faculty of Pharmacy, Sinai University, Kantara, Ismailia 41511, Egypt; 5 Graduate Institute of Natural Products, College of Pharmacy, Kaohsiung Medical University, Kaohsiung 80708, Taiwan; mickorinek@hotmail.com; 6 Department of Biotechnology, College of Life Science, Kaohsiung Medical University, Kaohsiung 80708, Taiwan; bhchen@kmu.edu.tw; 7 Graduate Institute of Natural Products, College of Medicine, Chang Gung University, Taoyuan 33302, Taiwan; d0901501@cgu.edu.tw; 8 Research Center for Chinese Herbal Medicine, Research Center for Food and Cosmetic Safety, and Graduate Institute of Health Industry Technology, College of Human Ecology, Chang Gung University of Science and Technology, Taoyuan 33302, Taiwan; 9 Department of Pharmaceutical Chemistry, Faculty of Pharmacy, The British University in Egypt (BUE), El-Sherouk City, Suez Desert Road, Cairo 11837, Egypt; amgad.albohy@bue.edu.eg (A.A.); basma.sabry@bue.edu.eg (B.S.A.); 10 Department of Medical Research, Kaohsiung Medical University Hospital, Kaohsiung 80708, Taiwan; 11 The Institute of Biomedical Sciences, National Sun Yat-sen University, Kaohsiung 80424, Taiwan; 12 Department of Biochemistry and Molecular Biology, College of Medicine, Chang Gung University, Taoyuan 33302, Taiwan; jimtong@mail.cgu.edu.tw; 13 State Key Laboratory of Natural and Biomimetic Drugs, Peking University, Beijing 100083, China; 14 Department of Anesthesiology, Chang Gung Memorial Hospital, Taoyuan 33302, Taiwan; 15 Department of Chemical Engineering, Ming Chi University of Technology, New Taipei City 24301, Taiwan; 16 Department of Pharmacognosy, Faculty of Pharmacy, Lokman Hekim University, Çankaya, Ankara 06510, Turkey

**Keywords:** *Aspergillus terreus*, butenolides, antiallergic, COVID-19 M^pro^, elastase

## Abstract

In December 2020, the U.K. authorities reported to the World Health Organization (WHO) that a new COVID-19 variant, considered to be a variant under investigation from December 2020 (VUI-202012/01), was identified through viral genomic sequencing. Although several other mutants were previously reported, VUI-202012/01 proved to be about 70% more transmissible. Hence, the usefulness and effectiveness of the newly U.S. Food and Drug Administration (FDA)-approved COVID-19 vaccines against these new variants are doubtfully questioned. As a result of these unexpected mutants from COVID-19 and due to lack of time, much research interest is directed toward assessing secondary metabolites as potential candidates for developing lead pharmaceuticals. In this study, a marine-derived fungus *Aspergillus terreus* was investigated, affording two butenolide derivatives, butyrolactones I (**1**) and III (**2**), a meroterpenoid, terretonin (**3**), and 4-hydroxy-3-(3-methylbut-2-enyl)benzaldehyde (**4**). Chemical structures were unambiguously determined based on mass spectrometry and extensive 1D/2D NMR analyses experiments. Compounds (**1**–**4**) were assessed for their in vitro anti-inflammatory, antiallergic, and in silico COVID-19 main protease (M^pro^) and elastase inhibitory activities. Among the tested compounds, only **1** revealed significant activities comparable to or even more potent than respective standard drugs, which makes butyrolactone I (**1**) a potential lead entity for developing a new remedy to treat and/or control the currently devastating and deadly effects of COVID-19 pandemic and elastase-related inflammatory complications.

## 1. Introduction

For more than a year now, since 2019, the whole world has been faced with the Coronavirus Disease 2019 (COVID-19) pandemic, believed to be caused by severe acute respiratory syndrome coronavirus 2 (SARS-CoV 2), a zoonotic viral infection that first emerged and was reported in Wuhan, China in December 2019 [1,2]. During this year, scientists from all around the globe set new horizons for collaborations to race against time to produce a dependable and a reliable vaccine. This mission was accomplished in December 2020 when the U.S. Food and Drug Administration (FDA) issued the first Emergency Use Authorization (EUA) for the Pfizer–BioNTech vaccine [3] and the United Kingdom approved the emergency use of the Oxford–AstraZeneca vaccine for the prevention of the COVID-19 in individuals 16 years of age or older [4]. However, simultaneously, two new viral variants were identified at two widely spaced localities in London, United Kingdom, designated as a variant under investigation of December 2020 (VUI-202012/01) and in Cape Town, South Africa, named 501.V2, that both shared a common worrying feature of being up to 70% more transmissible [5]. New variants are not the only ones evolved by SARS-CoV-2, but they are the ones that succeeded in adopting uncontrolled transmission among humans, forcing several countries to move back to strict lockdowns, social distancing and other infection control measures [5]. These two new variants also raised questions about the efficacy and effectiveness of the approved vaccines against them, which will take more time to figure out.

In other domains, more research efforts have been directed toward finding out other treatment alternatives to ease COVID-19 severity in vulnerable patients, especially acute respiratory distress symptoms mainly caused by neutrophil elastase (NE) enzyme, an intracellular enzyme stored in azurophilic granules of polymorphonuclear neutrophils (PMNs) that are a major component of human innate immunity [6,7]. Although NE’s main function is to devastate functional proteins of xenobiotics and/or pathogens of various origins, it has also been found to induce deleterious effects on the lungs as elastin-rich connective tissue affording pathologic edematous symptoms such as acute lung injury (ALI), acute respiratory distress syndrome (ARDS), or chronic obstructive pulmonary disorder (COPD) [7,8]. Moreover, NE contributes to the invasion of SARS-CoV-2 into host cells and plays a role in the COVID-associated ARDS, developed usually in the late stage of the COVID disease. Currently, sivelestat is the only approved NE inhibitor for the treatment of ARDS (in Korea and Japan) [9].

Fungi from various environments proved to be a prolific source producing a plethora of secondary metabolites with intriguing spectrum of biological activities and/or industrial applications [10]. The *Aspergillus* genus is among the most abundant fungal genera, comprising about 250 species, and is a major source contributing to the discovery of bioactive fungal metabolites [11]. *Aspergillus terreus* is a widely distributed fungus in diverse environments including extreme living conditions of high salinity [12], high temperature [13], high alkalinity [14], and drought [15]. Its ability to accommodate these extreme conditions stressed its probable evolution of gene clusters or regulatory mechanism to acclimatize these environments that may also induce the biosynthesis of a wide variety of fungal secondary metabolites including alkaloids, polyketides, peptides, terpenes, and lignans [11,16,17,18,19]. Butenolides (a rare type of lignans) and terretonins (meroterpenoids) are considered as typical metabolites of the genus *Aspergillus* that have exhibited a wide range of bioactivities such as antibacterial [20], cytotoxic [21], anti-inflammatory [22], antioxidant [23], and antiviral activities [24].

**Figure 1:**
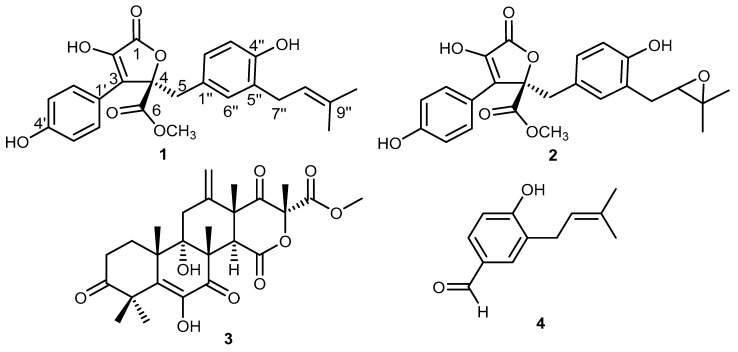
Chemical structures of **1**–**4**.

As part of our ongoing research directed toward exploring secondary metabolites for their relevant bioactivities, we investigated those obtained from a marine-derived fungus *Aspergillus terreus* for their in vitro anti-inflammatory, antiallergic, and in silico molecular modeling against the COVID-19 main protease (M^pro^) as a potential target for developing an antiviral drug. In this study, we report the isolation and identification of four different fungal metabolites (**1**–**4**) (Figure 1), their in vitro bioactivity assessment, and their in silico docking study results.

## 2. Results and Discussion

### 2.1. Isolation and Characterization of Main Secondary Metabolites in the Fungal Extract

A detailed chromatographic investigation of the solid rice culture extract from the cultivated fungal strain *Aspergillus terreus* derived from the marine annelide *Spirorbis* sp. was performed by applying different chromatographic procedures; that is, MS, 1D and 2D NMR spectral analyses, and comparing the obtained results with the reported literature. The obtained results afforded four different compounds.

Compound **1** was isolated as an orange-coloured amophous solid. Its UV spectrum revealed two absorption maxima (*λ*_max_) at 210 and 307 nm. The HRESIMS revealed the presence of two pseudomolecular ion peaks at *m*/*z* 447.12980 [M + Na]^+^ (calcd. for C_24_H_24_O_7_Na, 447.13092) and at *m*/*z* 423.12077 [M − H]^−^ (calcd. for C_24_H_23_O_7_, 423.12201). Hence, the molecular formula was established to be C_24_H_24_O_7_, indicating the existence of 13 degrees of unsaturation. The ^13^C NMR, DEPT, and HMQC spectra of **1** differentiated the presence of 11 quaternary carbons including two carbonyl groups (δ_C_ 170.1 and δ_C_ 169.8), three oxygenated olefinic carbons (δ_C_ 157.2, δ_C_ 153.2, and δ_C_ 144.9), five olefinic carbons (δ_C_ 137.7, 133.9, 128.8, 124.6, and 121.8), and one aliphatic quaternary carbon (δ_C_ 86.2). In addition, eight tertiary, two secondary, and three primary carbons were also distinguished. The ^1^H NMR spectrum of **1** clearly revealed the existence of two different aromatic systems, one recognized as 1,4-disubstituted phenyl and illustrated by two proton resonances each integrated for two protons at δ_H_ 6.90 (2H, d, *J* = 8.8 Hz) and at δ_H_ 7.61 (2H, d, *J* = 8.8 Hz). The second aromatic spin system was shown to be an 1,3,4-trisubstituted aromatic moiety as represented by three different proton resonances at δ_H_ 6.59 (1H, dd, *J* = 8.1, 2.0 Hz), δ_H_ 6.52 (1H, d, *J* = 8.1 Hz), and δ_H_ 6.51 (1H, d, *J* = 2.0 Hz). Moreover, the ^1^H NMR spectrum displayed three singlet methyl groups including one oxygenated methoxy group (δ_H_ 3.75/δ_C_ 53.7) and two olefinic methyl groups at δ_H_ 1.65 and δ_H_ 1.59 ppm directly connected to carbon peaks at δ_C_ 25.7 and δ_C_ 17.7 ppm, respectively. By comparing the obtained data with the reported fungal metabolites in the literature, they revealed a great agreement to butyrolactone I, a butenolide fungal metabolite first reported from *Aspergillus terreus* var. Africans IFO8355 [25,26,27].

Compound **2** was purified as a yellow-coloured amorphous solid showing absorption maxima (*λ*_max_) in its UV spectrum at 225 and 308 nm similar to those shown by **1**. The HRESIMS of **2** exhibited two pseudomolecular ion peaks at *m*/*z* 463.12586 [M + Na]^+^ (calcd. for C_24_H_24_O_8_Na, 463.12691) and at *m*/*z* 439.11538 [M − H]^−^ (calcd. for C_24_H_23_O_8_, 439.11654), indicating the molecular formula to be C_24_H_24_O_8_, differing by an additional oxygen atom compared with butyrolactone I (**1**) and similarly having 13 degrees of unsaturation. The ^1^H and ^13^C NMR spectra of **2** revealed a close similarity to **1**, except for the disappearance of the characteristic isoprenyl peaks and the existence of two oxygenated *sp^3^* carbons differentiated into one methine (δ_C_ 69.7) and one aliphatic quaternary carbon (δ_C_ 76.8) together with two singlet methyl groups at δ_H_ 1.21 (δ_C_ 24.8) and δ_H_ 1.24 (δ_C_ 22.0). The HMBC spectrum of **2** exhibited clear long-range correlations from the two singlet methyl groups to two carbons at δ_C_ 69.7 and δ_C_ 76.8, suggesting the replacement of isoprenyl moiety by an epoxy ring. By searching the reported literature, compound **2** was confirmed to be butyrolactone III [28,29].

Compound **3** was obtained as a creamy-coloured amorphous solid, with its UV spectrum showing two absorption maxima (*λ*_max_) at 220 and 278 nm. The molecular formula was determined to be C_26_H_32_O_9_ based on its HRESIMS spectrum, which disclosed the presence of two pseudomolecular ion peaks at *m*/*z* 489.2070 [M + H]^+^ (calcd. for C_26_H_33_O_9_, 489.2125), at *m*/*z* 511.1887 [M + Na]^+^ (calcd. for C_26_H_32_O_9_Na, 511.1944), and at *m*/*z* 487.1971 [M − H]^−^ (calcd. for C_26_H_31_O_9_, 487.1968), indicating the existence of 11 degrees of unsaturation. Both 1D and 2D NMR spectra of **3** revealed a similar pattern to those presented by terretonin, a meroterpenoid previously reported from *A. terreus* fungus [30,31].

Compound **4** was obtained as a red-coloured solid powder. Its ^13^C NMR spectrum revealed twelve distinct carbon resonances that can be differentiated through ^1^H NMR and HMQC experiment into four quaternary (δ_C_ 161.1, 132.2, 128.5, and 128.4), five tertiary including one aldehyde carbon at δ_C_ 191.1 s, together with three aromatic carbons (δ_C_ 130.7, δ_C_ 130.1, and δ_C_ 115.1) and an olefinic carbon at δ_C_ 121.9 [δ_H_ 5.29 (1H, td, *J* = 7.4, 1.5 Hz)]; one secondary carbon at δ_C_ 27.7 [δ_H_ 3.26 (2H, d, *J* = 7.4 Hz)]; in addition to two primary carbons at δ_C_ 25.5 [δ_H_ 1.71 (3H, d, *J* = 1.5 Hz)], and δ_C_ 17.6 [δ_H_ 1.68 (3H, d, *J* = 1.5 Hz)]. The ^1^H NMR spectrum in DMSO-*d_6_* revealed a clear 1,3,4-trisubstituted aromatic moiety through three proton resonances at δ_H_ 7.60 (1H, d, *J* = 8.2, 2.2 Hz), δ_H_ 7.58 (1H, d, *J* = 2.2 Hz), and δ_H_ 6.95 (1H, d, *J* = 8.2 Hz). In addition, it also showed two deshielded protons at δ_H_ 10.58 and δ_H_ 9.75 ppm ascribed for an aromatic hydroxyl group and an aldehyde moiety, respectively. By comparing the obtained 1D and 2D NMR data of **4** with the reported literature, it turned out to be identical to those reported for 4-hydroxy-3-(3-methylbut-2-enyl)benzaldehyde, a fungal metabolite first reported in 2012 from the root rotting pathogen *Heterobasidion occidentale* [32] and later reported from the fruits of *Narthecium ossifragum* [33].

Based on the previous reports about the activity of butyrolactones, they have been distinguished as potent inhibitors of cyclin-dependent kinases (CDKs) that play an important role in the occurrence of various diseases such as cancer, Alzheimer’s disease, Parkinson’s disease, stroke, diabetes, glomerulonephritis, and inflammation. Isolated butyrolactones (**1** and **2**) along with terretonin (**3**) and 4-hydroxy-3-(3-methylbut-2-enyl)benzaldehyde (**4**) were subjected to in vitro antiallergic, anti-inflammatory, anti-HCoV-229, and neutrophil elastase enzymatic assays. The viability assays towards the cells used in the tests were also performed.

### 2.2. Degranulation Assay in Mast Cells

The toxicity of isolated compounds was tested on RBL-2H3 cells up to 100 μM. The results revealed that all tested compounds were non-toxic as illustrated by the viability rate exceeding 90%. Isolated compounds (**1**–**4**) were then tested for their antiallergic activity via determining their inhibitory activities against A23187- and antigen-induced *β*-hexosaminidase release in RBL-2H3 cells. Calcium ionophore A23187 induces calcium transport into the mast cell membrane, whereas antigen (IgE plus DNP-BSA) acts via the FcεRI receptor, resembling a physiological condition. The attained results (Table 1) displayed that only butyrolactone I (**1**), among the tested compounds, showed moderate antiallergic activity, illustrated via inhibiting A23187- and antigen-induced degranulation with IC_50_ values of 39.7 and 41.6 μM, respectively, compared with azelastine as a standard antiallergic drug (34.5 and 35.5% at 10 μM, respectively). The obtained results are in accordance with and supported by the previously reported activity of **1** in alleviating ovalbumin-induced allergy symptoms via reducing the levels of histamine and mouse mast cell proteinases [34].

### 2.3. Human Neutrophil Viability, Elastase Release, and Elastase Enzymatic Assays

**Figure 2:**
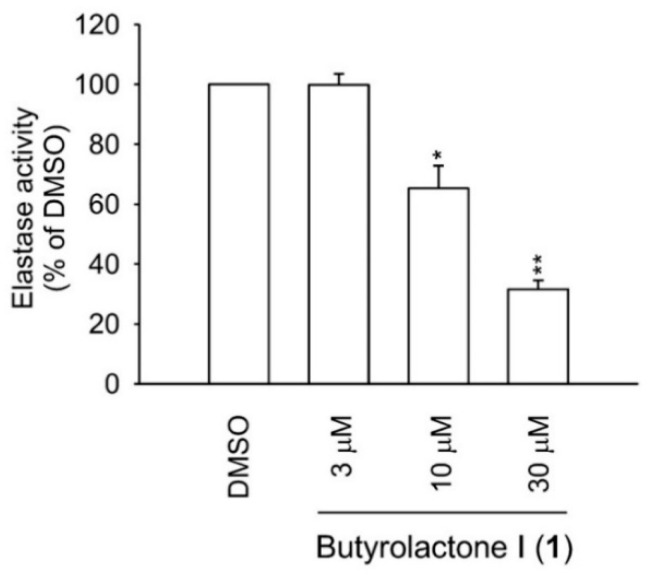
In vitro elastase inhibitory activity of butyrolactone I (**1**) in the cell-free system. The results are presented as mean ± S.E.M. (*n* = 3). * *p* < 0.05, ** *p* < 0.01 compared with the control (0.1% DMSO).

The results of an in vitro anti-inflammatory assay of compounds **1**–**4** (Table 2) revealed that only butyrolactone I (**1**) featured potent inhibitory activities against neutrophil elastase release (IC_50_ = 2.30 μM). Interestingly, butyrolacotone I (**1**) rather than III (**2**) exhibited significant activities more potent than genistein used as a standard drug (IC_50_ = 32.67 μM). Further, the cell viability assay based on lactate dehydrogenase release was performed to exclude toxic effects of **1** on human neutrophils. Both butyrolactone I (**1**) and III (**2**) were non-toxic to neutrophils (Table 2). Human neutrophil elastase plays a pivotal role in the development of several inflammatory symptoms including respiratory harmful effects accompanying several acute and chronic respiratory disorders [8]. In the cell-free system, butyrolacotone I (**1**) revealed a dose-dependent direct inhibitory effect on the enzymatic activity of elastase (Figure 2) with an IC_50_ value of 16.70 μM (Table 2). Based on these results, the anti-inflammatory effects of **1** were, at least partly, attributed to its interaction with elastase enzyme. Therefore, we performed the following in silico molecular modeling experiment to simulate and identify the interaction sites.

### 2.4. Molecular Docking Studies

**Figure 3:**
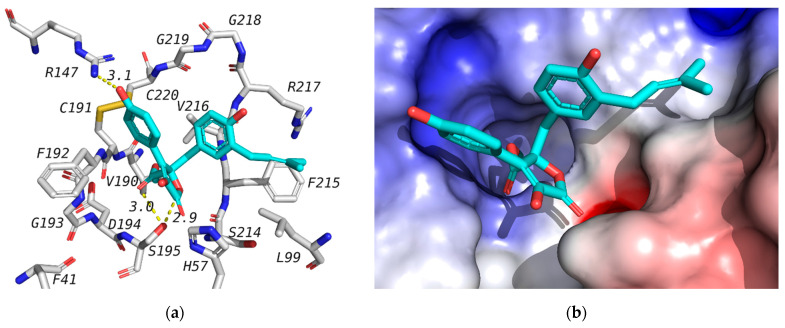
Docking of compound butyrolactone I (**1**) (blue) in the active site of human NE. (**a**) Interactions of **1** with amino acids in the active site. (**b**) Docking pose of **1**.

Docking studies were used to investigate the affinity of isolated compounds to the human neutrophil elastase (NE). The crystal structure of NE is available in the protein data bank (PDB) with the ID 1H1B co-crystalized with GW475151. Validation of the docking procedure was reported earlier, where the co-crystalized ligand was redocked in the active site with a docking score of −6.9 kcal/mol, and an RMSD of 1.317 between docked, and crystalized structures [35]. The co-crystalized ligand is known to form a hydrogen bond with Ser195 that is important for binding [36]. In this study, we have docked the isolated compounds (**1**–**4**) in the active site of the human NE. Out of the tested compounds, only butyrolactone I (**1**) has shown a docking score superior to that of the co-crystalized ligand (Table 3). All of tested compounds were found to form a hydrogen bond with Ser195 similar to GW475151, the co-crystalized ligand. It is worth mentioning here that **1** was found to inhibit human elastase in vitro with an IC_50_ of 16.70 µM (Table 2). The binding mode of compound **1** as well as its interaction with amino acids in the active site is shown in Figure 3. Butyrolactone III (**2**), which is a very similar structure, did not show similar results in either elastase assays (Table 3, no noticed inhibition at concentration up to 10 µM) or docking study. This might be attributed to the fact that the isoprene part of butyrolactone I (**1**) is docked in a hydrophobic side pocket, forming hydrophobic interactions with Phe21 and Leu99, as can be seen in Figure 3. This binding mode will not be favored for the epoxide ring of butyrolactone III (**2**), leading to a flipped alternative binding mode that is missing the key interaction with Ser195.

In addition to the human NE, we were also interested in investigating the potential binding and inhibitory activities of isolated compounds against SARS-CoV-2 main protease (M^pro^) owing to the current pandemic situation. The viral main protease is a key enzyme in the virus life cycle that has been the target for several investigations since the beginning of last year. The target crystal structure is available under PDB ID of 6LU7 co-crystalized with a peptide-like inhibitor called N3 [35]. It was found to interact with several amino acids in the active site, including Phe140, Gly143, His163, His164, Glu166, Gln189, and Thr190. Several research groups investigated synthetic and natural products for their inhibition of this target among other SARS-CoV-2 targets [36,37,38]. We have previously used the same target to investigate the potential inhibition of phytochemicals from the Jordanian hawksbeard [39]. We also reported validation of the same docking procedure through the docking of N3 co-crystalized ligand in the active site of 6LU7 [39]. The docking score of the co-crystalized ligand was found to be −7.1 kcal/mol.

**Figure 4:**
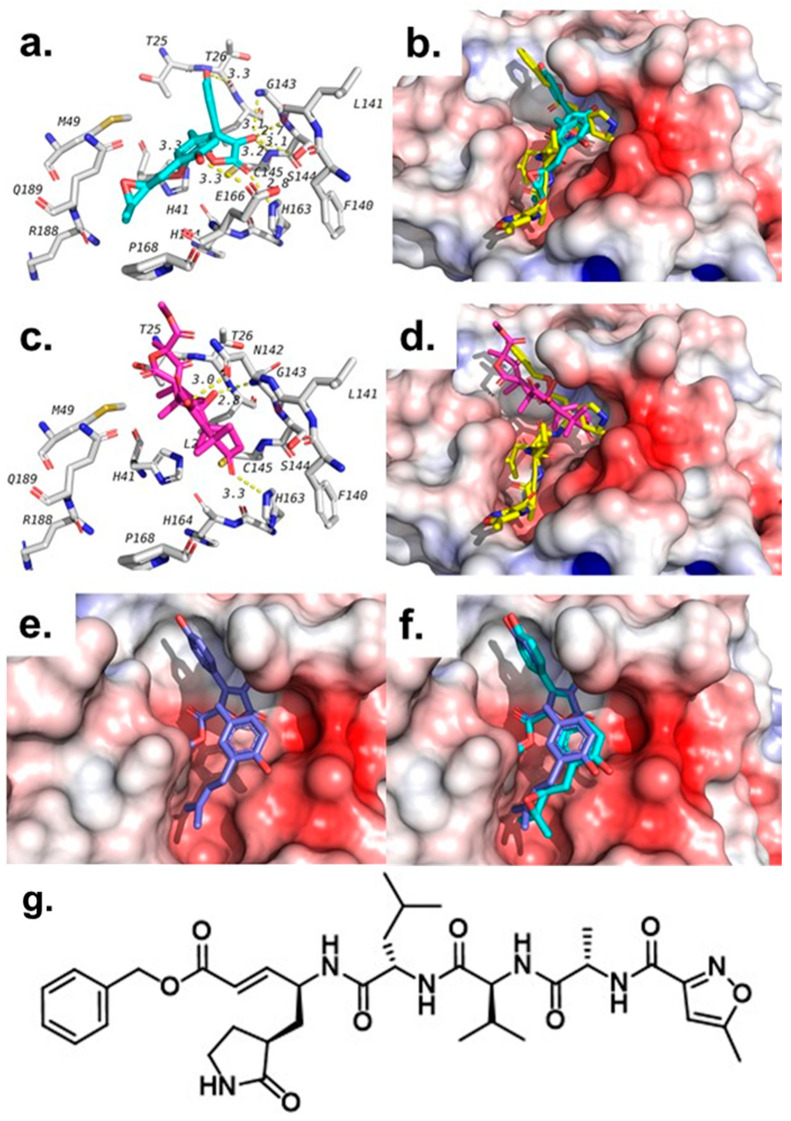
Poses and interactions of tested compounds in the active site of SARS-CoV-2 main protease (6LU7). (**a**) Interactions of butyrolactone III (**2**) (Cyan) in the active site. (**b**) Overlapping of butyrolactone III (**2**) with co-crystalized ligand N3 (yellow). (**c**) Interactions of terretonin (**3**) (pink) in the active site. (**d**) Overlapping of terretonin (**3**) with co-crystallized ligand N3 (yellow). (**e**) Binding pose of butyrolactone I (**1**). (**f**) Overlapping of the binding poses of butyrolactone I (**1**) and butyrolactone III (**2**). (**g**) Structure of the co-crystallized ligand (N3) in 6LU7 pdb file.

Among the tested compounds, butyrolactone III (**2**) and terretonin (**3**) have shown the best scores (−7.8 kcal/mol) compared with the co-crystalized ligand N3 (−7.1 kcal/mol), as shown in Table 3. Both compounds were found to bind in the same pocket where N3 binds, but each overlaps with slightly different parts of N3, as shown in Figure 4. In addition, both compounds were able to maintain two of the hydrogen bonds seen with N3, which include hydrogen bonds with Gly143 and His163.

Beside these two hydrogen bonds, both compounds were found to form other hydrogen bonds and hydrophobic interactions with residues in the active site of the SARS-CoV-2 main protease, as shown in Table 3 and Figure 4. In addition to these two compounds, butyrolactone I (**1**) has also shown a docking score that is better than that of the co-crystalized ligand (N3), but weaker than **2** and **3**. The docking pose of butyrolactone I (**1**) in the active site of the main protease is shown in Figure 4e. This docking pose is very similar, as expected, to the proposed binding pose of butyrolactone III (**2**), as seen in Figure 4f. Compound **4**, on the other hand, showed the lowest binding affinity (-5.6 kcal/mol) to SARS-CoV-2 main protease compared with the co-crystalized ligand N3. Furthermore, we performed an in vitro human coronavirus 229E (HCoV-229) assay to determine possible protective effects of compounds **1**–**4** (10 μM) against the HCoV-229 infection in Huh7 cells; however, none of the compounds exerted effects (see supplementary materials, Figure S1).

These results suggest a potential role of these isolated compounds in the inhibition of the SARS-CoV-2 main protease with a possible role in controlling the new virus and late stage of coronavirus-associated ARDS inflammation. The results also support the need for further investigation of these compounds as well as the natural products reservoir for new leads that could help us with our battle against the COVID-19 virus.

## 3. Materials and Methods 

### 3.1. General Experimental Procedures

A Perkin-Elmer-241 MC polarimeter was used for determining optical rotation. Chromatographic separation procedures were performed applying column chromatography with different stationary phases such as silica gel 60 M (0.04–0.063 mm) and Sephadex LH20. For screening purposes, ready-made silica gel 60 F_254_ TLC plates (Merck, Darmstadt, Germany) were used. For visualization purposes of TLC plates, UV light at 254 and 365 nm wavelengths was applied as a non-destructive technique or after spraying with anisaldehyde reagent and heating. Final purification of fractions was achieved using preparative HPLC (Agilent, Santa Clara, CA, U.S.A.) on Zorbax Eclipse XDB-C18 (Agilent technologies, Santa Clara, CA, U.S.A.) preparative column (9.4 mm × 250 mm, L × ID; 5 µm particle size) at a flow rate of 2 mL/min and UV screening detection at 210 to 330 nm. A standard gradient elution was applied using (MeOH, in Water): 0 min, 10% MeOH; 5 min, 10% MeOH; 40 min, 90% MeOH, with a flow rate of 1 mL/min. Each solvent ratio/flow timetable will be present for the compounds purified by HPLC. Preparative TLC separation was done using Flat Bottom TLC Chamber (Camag^®^, Muttenz, Switzerland). Silica gel 60 (0.04–0.063, Merck, Darmstadt, Germany) and Sephadex^®^ LH-20 (Sigma-Aldrich, St. Louis, MO, USA) were used for column chromatography and separation was monitored using normal phase silica gel precoated plates F_254_ (Merck, Darmstadt, Germany). An Agilent 600 MHz spectrometer (Santa Clara, CA, USA) was used for 1D (^1^H and ^13^C NMR) and 2D NMR spectra (chemical shifts in ppm). Chloroform-*d*, DMSO-*d*_6_ and methanol-*d*_4_ NMR solvents (Sigma Aldrich, Munich, Germany) were used to dissolve the isolated compounds. Supplementary materials of this study includes HPLC chromatograms, Mass, 1D, and 2D NMR spectra of isolated compounds along with NMR data for each compound in a tabulated form.

### 3.2. Sponge and Fungal Strain Material

The fungus *Aspergillus terreus* was separated from the annelide *Spirorbis* sp., which was collected by one of our co-authors (B.G.) from Marmara Sea, İstanbul, Turkey in July, 2018. For identification, this fungus was cultured on Sabouraud 4% dextrose agar (SDA, Merck, Germany) at room temperature for a week in an incubator (Nüve, Turkey). The fungus was identified as *Aspergillus terreus* (GenBank accession number MT273950) based on DNA amplification and ITS (internal transcribed spacer) sequencing data analysis, as reported previously in the literature. This fungal strain was deposited in the laboratory of the Department of Pharmacognosy, Faculty of Pharmacy, Ankara University (B.K.).

### 3.3. Fermentation, Extraction, and Isolation

The fungal strain was cultivated on a 100 mL solid rice medium prepared by autoclaving (100 g of rice and 100 mL of distilled water containing 3.5% artificial sea salt in a 60-piece 2000 mL Erlenmeyer flask). Fermentation continued for 30 days at room temperature away from light under static conditions.

To discontinue the fermentation process, ethyl acetate (EtOAc) (3 × 350 mL) was added to each flask to stop the growth of cells. After adding EtOAc, flasks were shaken for 12 h, then filtered, and EtOAC filtrate was pooled and evaporated under reduced pressure until yielding a solid residue (3.7 g). The crude extract was then partitioned between *n*-hexane and 90% aqueous MeOH by liquid–liquid fractionation, where both fractions were collected and dried up under vacuum. The aqueous 90% MeOH phase (1.3 g) was subjected to vacuum liquid chromatography (VLC) using silica gel 60 as a stationary phase and the mobile phase started with *n*-hexane/EtOAc (30:70) followed by a gradient elution development of DCM/MeOH (100:0, 90:10, 80:20, 70:30, 60:40, 50:50, 30:70, 10:90, 0:100), respectively, affording ten fractions (AT-1~AT-10).

All obtained fractions were subjected to TLC and analytical HPLC procedures. Fraction AT-2 was chosen for further preparative TLC and HPLC purification procedures. Fraction AT-2 (272 mg), eluted with DCM/MeOH (100:0), was applied on column chromatography using silica gel (Qingdao Haiyang Chemical HG/T2354-92) as stationary phase and petroleum ether/EtOAc as mobile phase at ratios of 4:1, 3:1, 2:1, and 1:1, each 300 mL, respectively, yielding **1** (4.4 mg), **2** (5.3 mg), and **4** (1.8 mg). The subfraction of AT-4 (78 mg) from silica column application was applied on column chromatography using silica gel as the stationary phase and petroleum ether/EtOAc as the mobile phase at ratios of 4:1, 3:1, 2:1, and 1:1, each 200 mL, followed by preparative HPLC for final purification to yield **3** (2.0 mg).

*Butyrolactone I* (**1**). Orange-coloured amophous solid; ${\left[\alpha \right]}_{D}^{20}$ +91.0° (*c* 0.02, MeOH); UV (MeOH) *λ*_max_ 210 and 307 nm; ^1^H and ^13^C NMR, see supplementary material Table S1; HRESIMS *m*/*z* 447.12980 [M + Na]^+^ (calcd. for C_24_H_24_O_7_Na, 447.13092) and at *m*/*z* 423.12077 [M − H]^−^ (calcd. for C_24_H_23_O_7_, 423.12201).

*Butyrolactone III* (**2**). Yellow-coloured amorphous solid; ${\left[\alpha \right]}_{D}^{20}$ +76.0° (*c* 0.02, MeOH); UV (MeOH) *λ*_max_ 225 and 308 nm; ^1^H and ^13^C NMR, see supplementary material Table S2; HRESIMS *m*/*z* 463.12586 [M + Na]^+^ (calcd. for C_24_H_24_O_8_Na, 463.12691) and at *m*/*z* 439.11538 [M − H]^−^ (calcd. for C_24_H_23_O_8_, 423.11654).

*Terretonin* (**3**). Creamy-coloured amorphous solid; ${\left[\alpha \right]}_{D}^{20}$ −112.4° (*c* 0.02, MeOH); UV (MeOH) *λ*_max_ 220 and 278 nm; ^1^H and ^13^C NMR, see supplementary material Table S3; HRESIMS *m*/*z* 489.2070 [M + H]^+^ (calcd. for C_26_H_33_O_9_, 489.2125), *m*/*z* 511.1887 [M + Na]^+^ (calcd. for C_26_H_32_O_9_Na, 511.1944), and *m*/*z* 487.1971 [M − H]^−^ (calcd. for C_26_H_31_O_9_, 487.1968).

*4-Hydroxy-3-(3-methylbut-2-enyl)benzaldehyde* (**4**). Red-coloured solid powder; ^1^H and ^13^C NMR, see supplementary material Table S4.

### 3.4. Degranulation Assay and MTT Cell Viability Assay in Mast Cells

The mucosal mast-cell-derived rat basophilic leukemia cells (RBL-2H3) were purchased from Bioresource Collection and Research Center (Hsin-Chu, Taiwan). The cells were cultured in DMEM containing 10% FBS, 100 U/mL penicillin, and 100 μg/mL streptomycin in 10 cm cell culture dishes at 37 °C in a humidified chamber with 5% CO_2_ in air. The level of degranulation in RBL-2H3 cells was evaluated using *β*-hexosaminidase release assay induced by A23187 or antigen as reported before with some modifications [40]. Briefly, the cells were seeded in a 96-well plate (2 × 10^4^ cells/well, for the A23187-induced assay) or a 48-well plate (3 × 10^4^ cells/well, for the antigen-induced assay) overnight. The cells for the antigen-induced assay were sensitized with anti-DNP IgE (0.5 μg/mL; Sigma) during seeding overnight. RBL-2H3 cells were then treated with the samples (0.5, 5, and 50 μM) for 30 min in Tyrode’s buffer with a maximal DMSO dose of 0.5%. For the A23187-induced assay, the cells were activated by addition of A23187 (final concentration 0.5 μM), while cells for the antigen-induced assay were activated by the addition of DNP-BSA (final concentration 100 ng/mL) for 30 min. Azelastine (10 μM) served as the positive control. The amount of *β*-hexosaminidase was detected using the method utilizing p-NAG as the substrate according to the procedure described before [41].

The viability of the RBL-2H3 cells in the presence of the samples (10 and 100 μM) was determined using the methylthiazole tetrazolium (MTT) assay according to a previous method [41].

### 3.5. Elastase Release Assay and Lactate Dehydrogenase (LDH) Viability Assay by Human Neutrophils

The human neutrophils were obtained from venous blood of healthy adult volunteers (20–30 years old) following the reported procedure [42]. Elastase release by the activated neutrophils was determined using elastase substrate *(N*-methoxysuccinyl-Ala-Ala-Pro-Val-*p*-nitroanilide) according to the previous methodology [42]. The tested samples’ concentration was 1 to 10 μM and the total incubation time in fMLF/CB-induced cells was 15 min. Genistein was used as the positive control. Cytotoxicity test was performed based on the release of LDH stored in the cytoplasm out of the cells [43]. Briefly, preheated (37 °C, 5 min, 1 mM CaCl_2_) human neutrophils (6 × 10^5^ cells·mL^−1^) were incubated with test compounds for 15 min. Total LDH release control was represented as completely lysed cells by 0.1% of Triton X-100 solution incubated with cells for 30 min. The cells were centrifuged at 4 °C for 200× *g* for 8 min, and LDH reagent was added to supernatant and reacted at room temperature for 30 min in the dark. The absorbance was then measured at 492 nm, and the LDH release was calculated and compared to the total LDH release set as 100%.

### 3.6. Determination of Elastase Enzymatic Activity

The compounds were further tested for direct inhibition of elastase enzymatic activity [43]. The neutrophil suspension (6 × 10^5^ cells mL^−1^) was preheated for 5 min in the presence of CaCl_2_ (1 mM) at 37 °C. Priming agent CB (1.5 μg mL^−1^) was added for 2 min, followed by fMLF (0.1 μM) for 20 min to release the elastase from the cells. After centrifugation at 1000 g for 5 min at 4 °C, the supernatant containing elastase was preheated at 37 °C for 5 min, and the test compounds were added. Then, 0.1 mM of substrate methoxysuccinyl-Ala-Ala-Pro-Val-*p*-nitroanilide was added for 10 min. The effect of the compounds on elastase enzymatic activity was quantified by measuring the absorbance at 405 nm.

### 3.7. Molecular Modeling Studies

Docking study was done using the procedure we reported and validated earlier [39]. Tested compounds were downloaded from Pubchem (www.pubchem.ncbi.nlm.nih.gov, accessed on 10 May 2021) or built from the 2D structures. Ligands and proteins were prepared as reported earlier [44]. Docking analysis and image preparation were done using PyMol. The proposed binding mode of the isolated compounds with neutrophil elastase (NE) and SARS-CoV-2 main protease (M^pro^) was studied using Autodock Vina and a method similar to what we reported earlier [39]. Here, crystal structures of NE (PDB ID:1H1B) and SARS-CoV-2 M^pro^ (PDB ID: 6LU7) were used. Prepared and co-crystalized ligands were docked in a grid box in the active site (25 × 25 × 25 Å^3^, centered on co-crystalized ligand) using exhaustiveness of 16. For each ligand, the top nine binding poses were ranked according to their binding affinities and the predicted binding interactions were analyzed. The pose with the best binding affinity and binding mode similar to co-crystalized ligand was reported.

### 3.8. Coronavirus 229E Assay

The protective effects of the samples against human coronavirus 229E (HCoV-229) were determined similarly to the previously described method [45]. Huh7 cells (human liver carcinoma cell line) were infected with 9TCID50 (median tissue culture infectious dose) of each coronavirus 229E in the presence or absence of the compounds or vehicle. After incubation at 33 °C for 6 days, the surviving cells were then stained with MTT (3-[4.5-dimethylthiazol-2-yl]-2,5-diphenyl tetrazolium bromide). The percentage of surviving cells was then calculated.

## 4. Conclusions

Two butenolides, butyrolactons I (**1**) and III (**2**), along with one meroterpenoid, terretonin (**3**), and a prenylated hydroxybenzaldehyde derivative (**4**) were isolated from a marine-derived fungus *Aspergillus terreus*. Interestingly, butyrolactone I (**1**) revealed significant in vitro antiallergic, anti-inflammatory, and antielastase activity. These results were supported by molecular docking studies that also exhibited a possible potential role of **1** for inhibiting SARS-CoV-2 main protease, an essential enzyme for producing the viral functional proteins. These results shed more light on butyrolactone I (**1**) and other butenolide derivatives as potential candidates for developing lead compounds that may pave the way for producing new pharmaceuticals against SARS-CoV-2 and/or its pathological effects, in particular, ARDS, granting additional time for the immune system to fight for the patient’s life.

## Figures and Tables

**Table 1:** Inhibitory activity of butyrolactone I (**1**) on A23187- and antigen-induced degranulation.

Compound.	Cell Viability, RBL-2H3 ^a^	Inhibition of A23187-Induced Degranulation ^b^	Inhibition of Antigen-Induced Degranulation ^c^
	% (100 μM)	IC_50_ (μM) ^d^	IC_50_ (μM) ^d^
Butyrolactone I (**1**)	>90%	39.7 ^e^	41.6 ^f^
Butyrolactone III (**2**)	>90%	>100	>100
Terretonin (**3**)	>90%	>100	>100
4-Hydroxy-3-(3-methylbut-2-enyl)benzaldehyde (**4**)	>90%	>100	>100

^a^ The cytotoxicity of samples to RBL-2H3 was evaluated using MTT viability assay. The results are presented as mean (*n* = 3) compared with untreated control (DMSO). All samples were nontoxic towards RBL-2H3 cells. ^b^ Azelastine (10 μM) was used as a positive control and inhibited 34.5 ± 2.5% of A23187-induced degranulation. The inhibition of degranulation was assessed by A23187-induced *β*-hexosaminidase release in RBL-2H3 cells. The results are presented as mean ± S.E.M. (*n* = 3). ^c^ Azelastine (10 μM) was used as a positive control and inhibited 35.5 ± 8.1% of antigen-induced degranulation. The inhibition of degranulation was assessed by antigen-induced *β*-hexosaminidase release in RBL-2H3 cells. The results are presented as mean ± S.E.M (*n* = 3). ^d^ Concentration necessary for 50% inhibition (IC_50_). ^e^ Butyrolactone I (**1**) showed dose-dependent inhibition of A23187-induced degranulation with 26.0 ± 2.4% (10 μM) and 98.7 ± 0.7% inhibition at 100 μM. ^f^ Butyrolactone I (**1**) showed dose-dependent inhibition of antigen-induced degranulation with 30.0 ± 3.3% (10 μM) and 87.0 ± 2.4% inhibition at 100 μM. >100, not active (insignificant inhibition of degranulation at 100 μM, below 20%).

**Table 2:** Effects of the isolated compounds from the marine-derived fungus *Aspergillus terreus* on elastase release, viability, and elastase enzymatic activity in vitro.

Compound	Elastase Release in Human Neutrophils *^a^*	Cell viability, Human Neutrophils *^c^*	Elastase Enzymatic Activity (Cell-Free) *^d^*
	IC_50_ (μM) *^b^*	% (10 μM)	IC_50_ (μM) *^b^*
Butyrolactone I (**1**)	2.30 ± 0.27	94.13 ± 2.31	16.70 ± 2.64
Butyrolactone III (**2**)	>10	98.25 ± 1.77	>30
Terretonin (**3**)	>10	n.t.	n.t.
4-Hydroxy-3-(3-methylbut-2-enyl)benzaldehyde (**4**)	>10	n.t.	n.t.

*^a^* Inhibition of elastase release in fMLF/cytochalasin B (CB)-induced human neutrophils. Genistein inhibited elastase release with an IC_50_ value 32.67 ± 1.45. *^b^* Concentration necessary for 50% inhibition (IC_50_). The results are presented as mean ± S.E.M. (*n* = 3). * *p* < 0.05, ** *p* < 0.01, *** *p* < 0.001 compared with the control (0.1% DMSO). *^c^* Percentage of cell viability (%) at 10 μM. The results are based on the lactate dehydrogenase release and presented as mean ± S.E.M. (*n* = 3). *^d^* Sivelestat was used as a positive control and inhibited elastase enzyme with an IC_50_ value 17.92 ± 4.66 nM. n.t.: not tested.

**Table 3:** Docking results of tested compounds in the active sites of human NE (1H1B) and SARS-CoV-2 main protease (6LU7). Amino acids’ interactions with both the co-crystalized ligands and tested ligands are shown bold.

Ligand	1H1B (Elastase)		6LU7 (M^pro^)	
	Binding Affinity (kcal/mol)	Interacting Residues	Binding Affinity (kcal/mol)	Interacting Residues
Butyrolactone I (**1**)	−7.3	**Ser195**-Arg147	−7.3	**Gly143**-Ser144- **His163**-**Glu166**
Butyrolactone III (**2**)	−6.7	**Ser195**-Arg147	−7.8	Thr26- Leu141-**Gly143**- Ser144- Cys145- **His163**- **Glu166**
Terretonin (**3**)	−6.7	**Ser195**- Val216	−7.8	Asn142- **Gly143**- **His163**
4-Hydroxy-3-(3-methylbut-2-enyl)benzaldehyde (**4**)	−5.1	**Ser195**- Cyc191	−5.6	Leu141-**Gly143**-Ser144-Cys145- **Glu166**
1H1B-Ligand	−6.9	**Ser195**	--	--
6lu7-Ligand	--	--	−7.1 (3rd pose)	Phe140, Gly143, His163, His164, Glu166, Gln189, Thr190

## Data Availability

Data are available upon request from authors.

## Supplementary Materials

The following are available online, Figure S1: Human coronavirus 229E (HCoV-229E) protective activity, Table S1: 1H and 13C NMR Data of butyrolactone I (**1**), Table S2: 1H and 13C NMR Data of butyrolactone III (**2**), Table S3: 1H and 13C NMR Data of terretonin (**3**), Table S4: 1H and 13C NMR Data of 4-hydroxy-3-(3-methylbut-2-enyl)benzaldehyde (**4**).

## Sample Availability

Samples of compounds (**1** and **2**) are available from the authors upon request.
