# Supplementary material for: Anti-Inflammatory, Antiallergic, and COVID-19 Main Protease (Mpro) Inhibitory Activities of Butenolides from a Marine-Derived Fungus Aspergillus terreus
Source: Molecules. 2021 Jun 2;26(11):3354. doi: 10.3390/molecules26113354 (PMC8199578; doi:10.3390/molecules26113354)
Supplement: Supplementary file 1 [file molecules-26-03354-s001.zip › molecules-1241619-supplementary.pdf]

Supplementary Materials for

Anti-Inflammatory, Antiallergic and COVID-19 Main Protease (M^{pro}) Inhibitory Activities of Butenolides from a Marine-Derived Fungus *Aspergillus terreus*

Ibrahim Seyda Uras [1,2,†], Sherif S. Ebada [3,4,*,†], Michal Korinek [5,6,7,8], Amgad Albohy [9], Basma S. Abdulrazik [9], Yi-Hsuan Wang [7], Bing-Hung Chen [6,10,11], Jim-Tong Horng [12], Wenhan Lin [13,*], Tsong-Long Hwang [7,8,14,15,*], and Belma Konuklugil [1,16*]

1 Department of Pharmacognosy, Faculty of Pharmacy, Ankara University, 06560 Ankara, Turkey; isuras@ankara.edu.tr
2 Department of Pharmacognosy, Faculty of Pharmacy, Agri Ibrahim Cecen University, 04100 Agri, Turkey
3 Department of Pharmacognosy, Faculty of Pharmacy, Ain Shams University, Abbasia, 11566 Cairo, Egypt
4 Department of Pharmacognosy, Faculty of Pharmacy, Sinai University, Kantara, Ismailia, Egypt
5 Graduate Institute of Natural Products, College of Pharmacy, Kaohsiung Medical University, Kaohsiung, Taiwan; mickorinek@hotmail.com
6 Department of Biotechnology, College of Life Science, Kaohsiung Medical University, Kaohsiung, Taiwan; bhchen@kmu.edu.tw
7 Graduate Institute of Natural Products, College of Medicine, Chang Gung University, Taoyuan, Taiwan; d0901501@cgu.edu.tw
8 Research Center for Chinese Herbal Medicine, Research Center for Food and Cosmetic Safety, and Graduate Institute of Health Industry Technology, College of Human Ecology, Chang Gung University of Science and Technology, Taoyuan, Taiwan
9 Department of Pharmaceutical Chemistry, Faculty of Pharmacy, The British University in Egypt (BUE), El-Sherouk City, Suez Desert Road, Cairo 11837, Egypt; amgad.albohy@bue.edu.eg (A.A.); basma.sabry@bue.edu.eg (B.S.A.)
10 Department of Medical Research, Kaohsiung Medical University Hospital, Kaohsiung, Taiwan
11 The Institute of Biomedical Sciences, National Sun Yat-sen University, Kaohsiung, Taiwan
12 Department of Biochemistry and Molecular Biology, College of Medicine, Chang Gung University, Taoyuan, Taiwan; jimtong@mail.cgu.edu.tw
13 State Key Laboratory of Natural and Biomimetic Drugs, Peking University, Beijing 100083, China
14 Department of Anesthesiology, Chang Gung Memorial Hospital, Taoyuan, Taiwan
15 Department of Chemical Engineering, Ming Chi University of Technology, New Taipei City 24301, Taiwan
16 Department of Pharmacognosy, Faculty of Pharmacy, Lokman Hekim University, 06510 Çankaya, Ankara, Turkey
† These authors equally contributed to the work in this study.
* Correspondence: sherif_elsayed@pharma.asu.edu.eg (S.S.E.); htl@mail.cgu.edu.tw (T.-L.H.); whlin@bjmu.edu.cn (W.L.); konuklug@pharmacy.ankara.edu.tr (B.K.) Tel.: +20-2405-1180 (S.S.E.); Fax: +20-2405-1107 (S.S.E.)

† These authors equally contributed to the work in this study.
* Correspondence: sherif_elsayed@pharma.asu.edu.eg (S.S.E.); htl@mail.cgu.edu.tw (T.-L.H.); whlin@bjmu.edu.cn (W.L.); konuklug@pharmacy.ankara.edu.tr (B.K.) Tel.: +20-2405-1180 (S.S.E.); Fax: +20-2405-1107 (S.S.E.)

ABSTRACT

In December 2020, the UK authorities reported to the WHO that a new COVID-19 variant, considered to be a Variant Under Investigation from December 2020 (VUI-202012/01) was identified through viral genomic sequencing. However, several other mutants were previously reported, VUI-202012/01 proved to be about 70% more transmissible. Hence, the usefulness and effectiveness of the newly FDA-approved COVID-19 vaccines against these new variants are doubtfully questioned. As a result of these unexpected mutants from COVID-19 and due to lack of time, much research interest is directed toward assessing secondary metabolites as potential candidates for developing lead pharmaceuticals. In this study, a marine-derived fungus *Aspergillus terreus* was investigated affording two butenolide derivatives, butyrolactones I (**1**) and III (**2**), a meroterpenoid, terretonin (**3**) and 4-hydroxy-3-(3-methylbut-2-enyl)benzaldehyde (**4**). Chemical structures were unambiguously determined based on mass spectrometry and extensive 1D/2D NMR analyses experiments. Compounds (**1**-**4**) were assessed for their *in vitro* anti-inflammatory, antiallergic and *in silico* COVID-19 main protease (M^{pro}) and elastase inhibitory activities. Among the tested compounds, only **1** revealed significant activities comparable or even more potent than respective standard drugs that makes butyrolactone I (**1**) a potential lead entity for developing a new remedy to treat and/or control the currently devastating and deadly effects of COVID-19 pandemic and elastase-related inflammatory complications.

Keywords: *Aspergillus terreus*; butenolides; antiallergic; COVID-19 M^{pro}; elastase

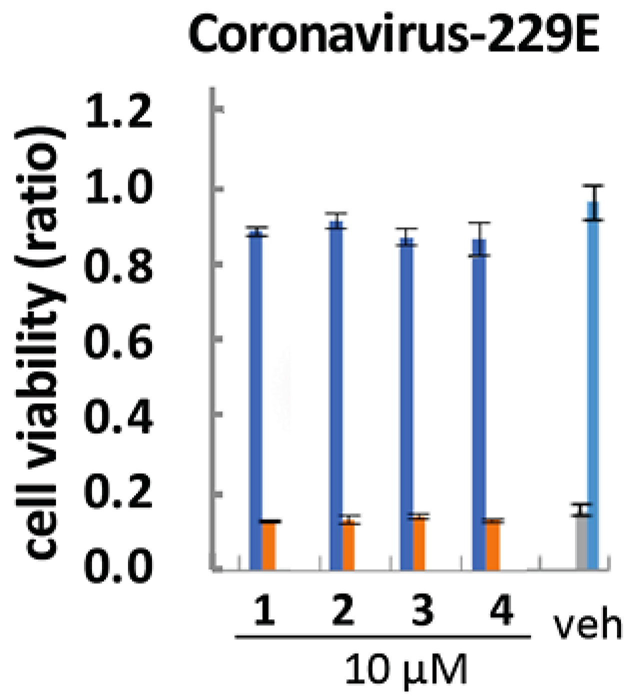

Figure S1. Human coronavirus 229E (HCoV-229E) protective activity. The cells infected by HCoV-229E were treated with the compounds (orange) or vehicle (gray). The difference would indicate protective effects against HCoV-229E infection. The uninfected cells were treated with the compounds (dark blue) or vehicle only (cyan), serving as control for toxicity of the samples and vehicle. Veh; vehicle

Table S1. ^{1}H and ^{13}C NMR Data of butyrolactone I (**1**).

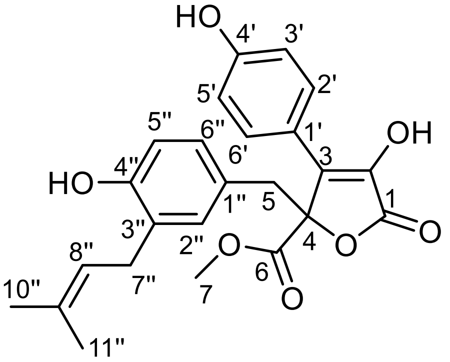

Butyrolactone I (**1**)

Butyrolactone (**1**) in chloroform-*d*, 400 MHz		
#	δ_H (*J*[Hz])	δ_C, type
1		169.8, C
2		144.9, C
3		137.7, C
4		86.2, C
5	3.53, d (15.0)<br>3.47, d (15.0)	38.7, CH_2
6		170.1, C
7	3.75, s (3H)	53.7, CH_3
1'		121.8, C
2'	7.61, d (8.8)	129.6, CH
3'	6.90, d (8.8)	116.1, CH
4'		157.2, C
5'	6.90, d (8.8)	116.1, CH
6'	7.61, d (8.8)	129.6, CH
1"		124.6, C
2"	6.51, d (2.0)	131.8, CH
3"		128.8, C
4"		153.2, C
5"	6.52, d (8.1)	115.1, CH
6"	6.59, dd (8.1, 2.0)	129.2, CH
7"	3.12, d (7.2, 2H)	28.7, CH_2
8"	5.08, td (6.7, 5.7, 3.8)	121.8, CH
9"		133.9, C
10"	1.65, s (3H)	25.7, CH_3
11"	1.59, s (3H)	17.7, CH_3

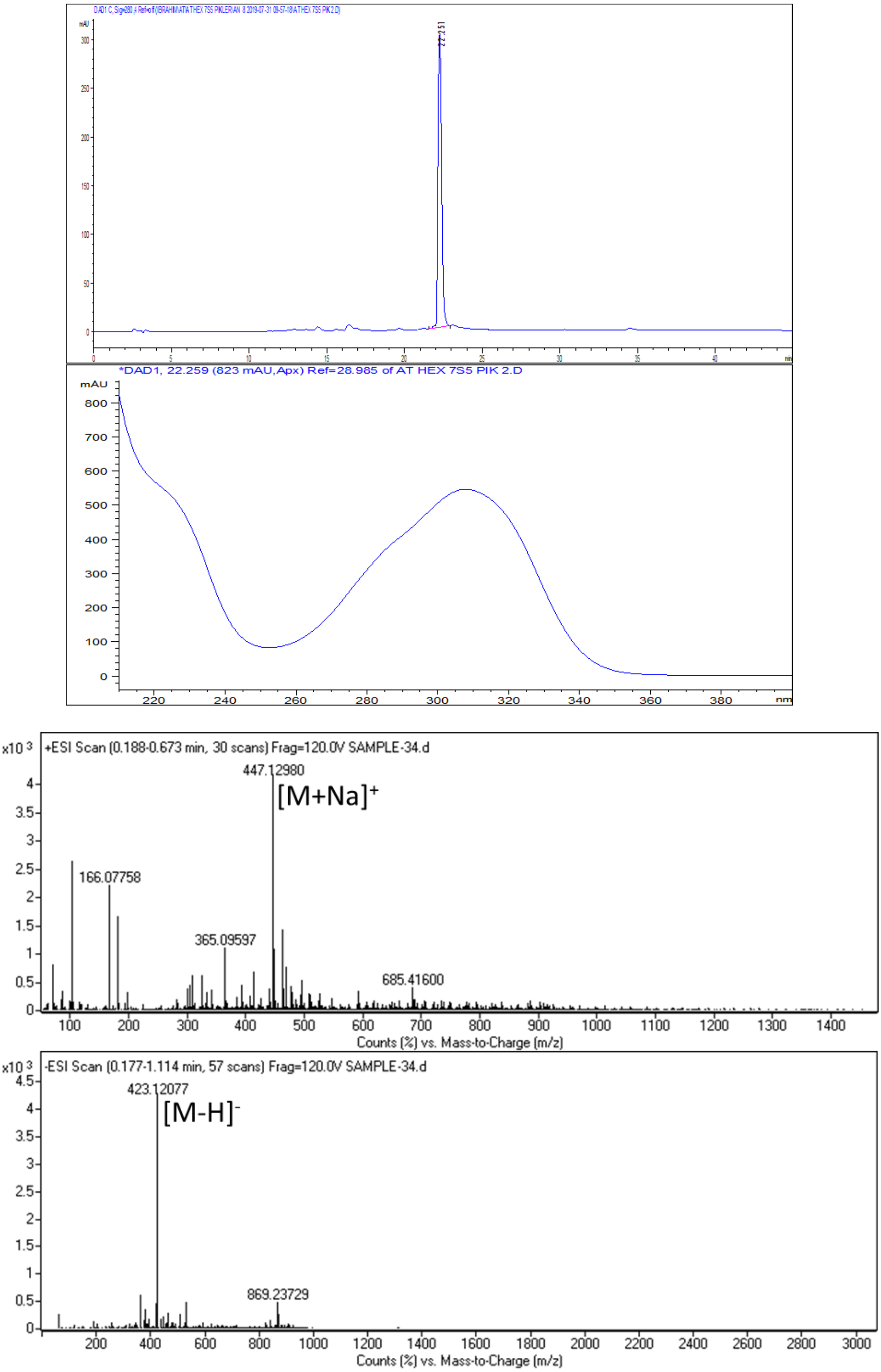

Figure S2. HPLC chromatogram and HRESIMS of **1**

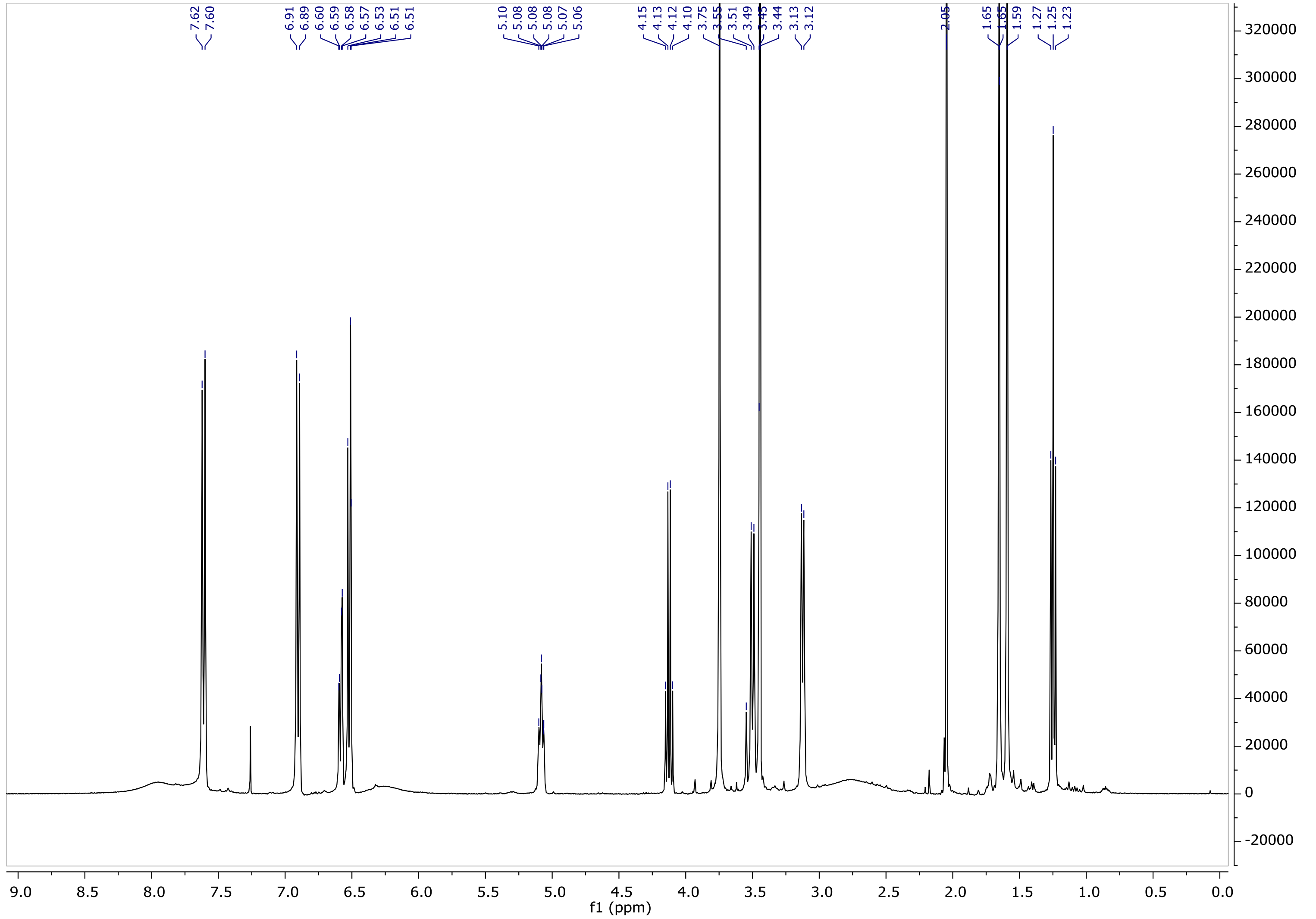

Figure S3. ^{1}H-NMR spectrum of **1** in chloroform-*d*

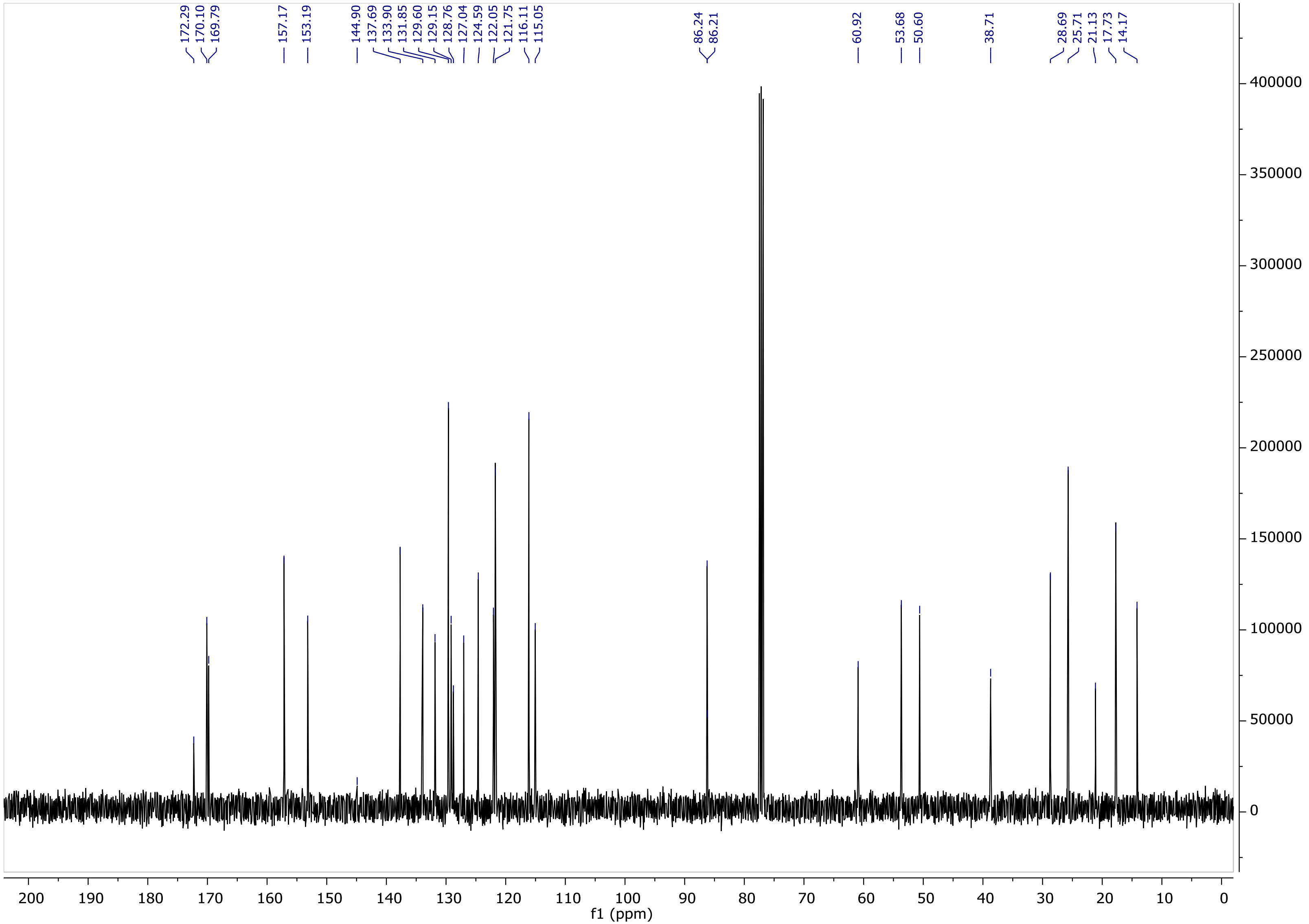

Figure S4. ^{13}C-NMR spectrum of **1** in chloroform-*d*

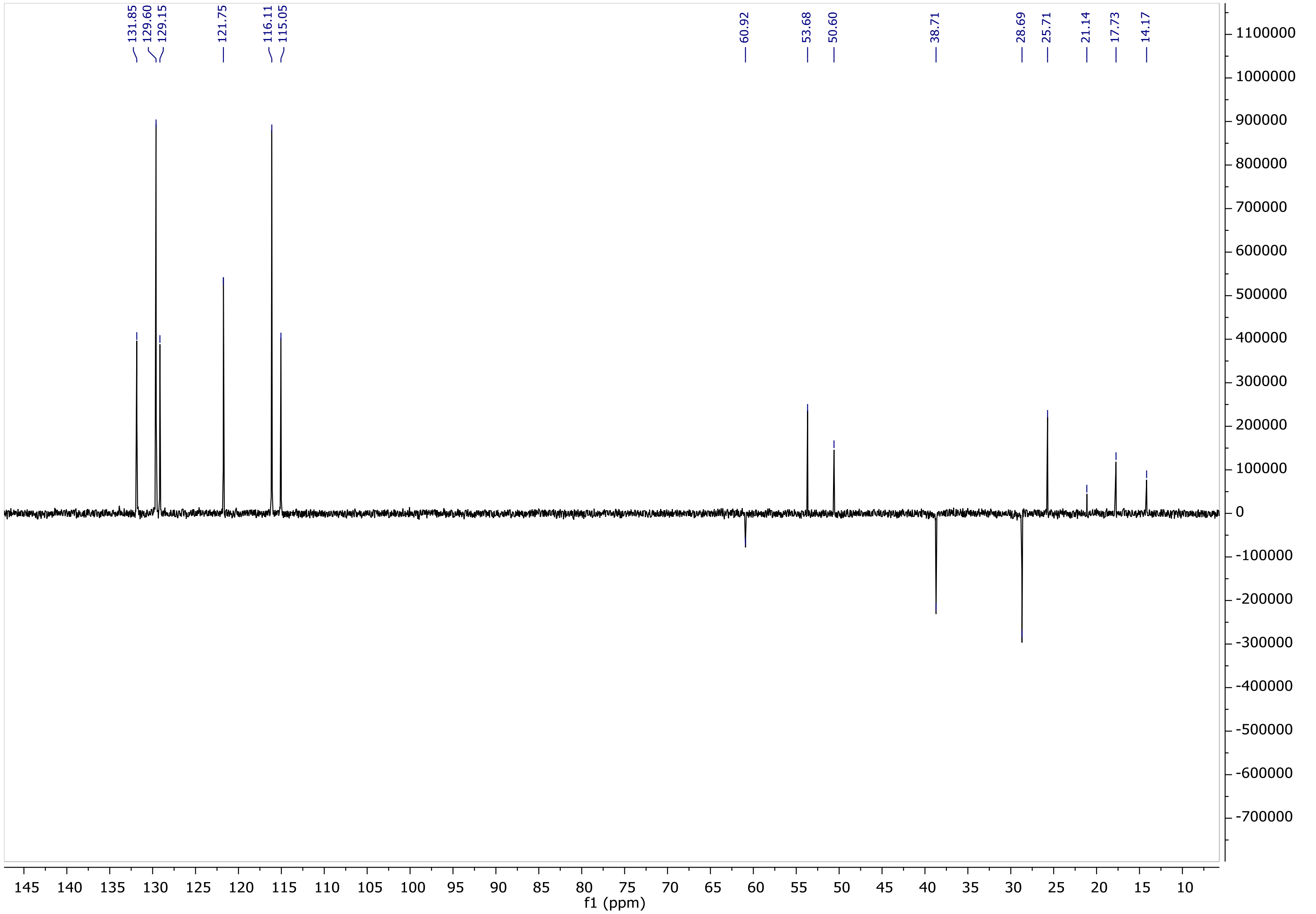

Figure S5. DEPT spectrum of **1** in chloroform-*d*

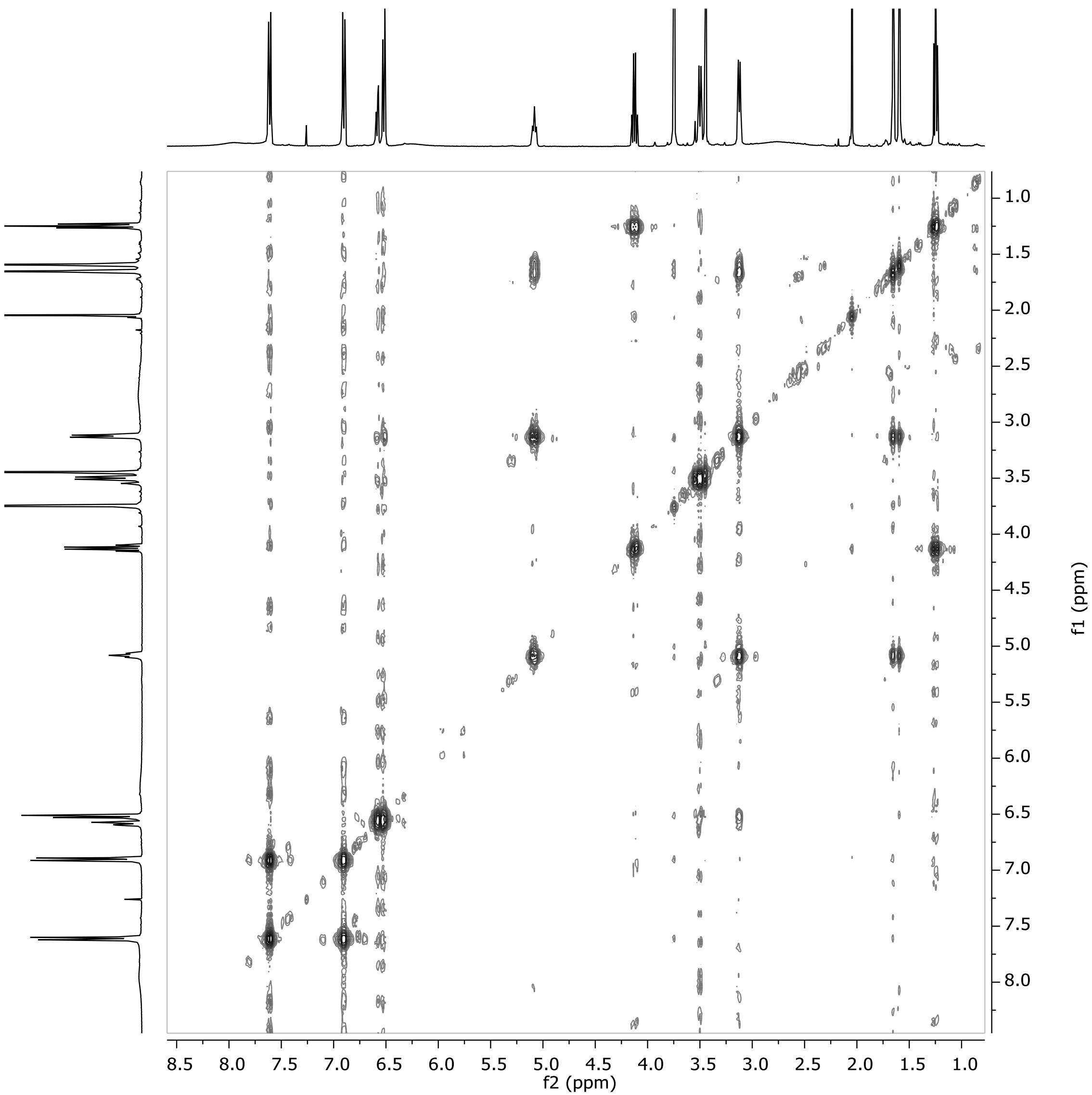

Figure S6. ^{1}H–^{1}H COSY spectrum of **1** in chloroform-*d*

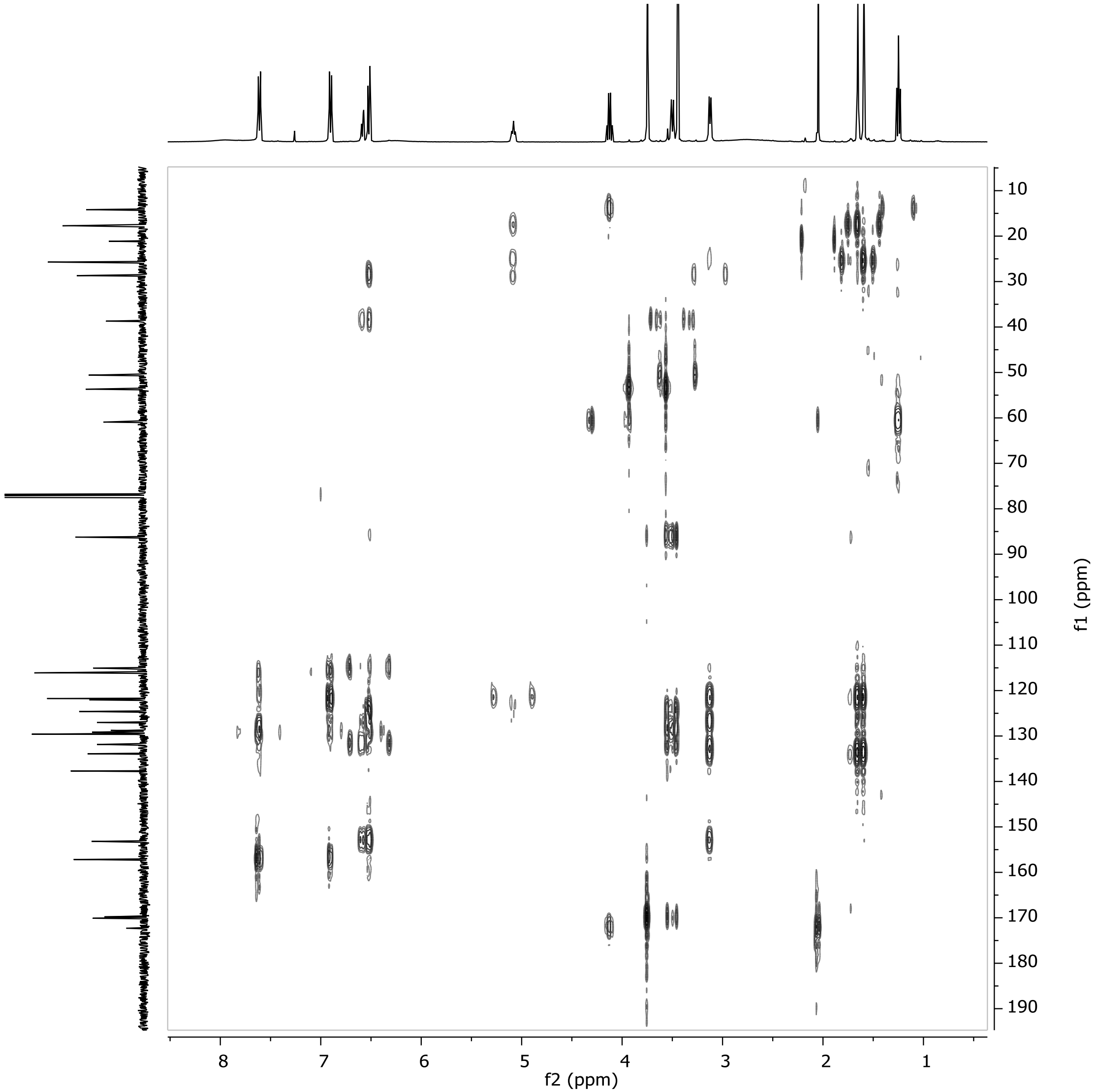

Figure S7. *g*HMBC spectrum of **1** in chloroform-*d*

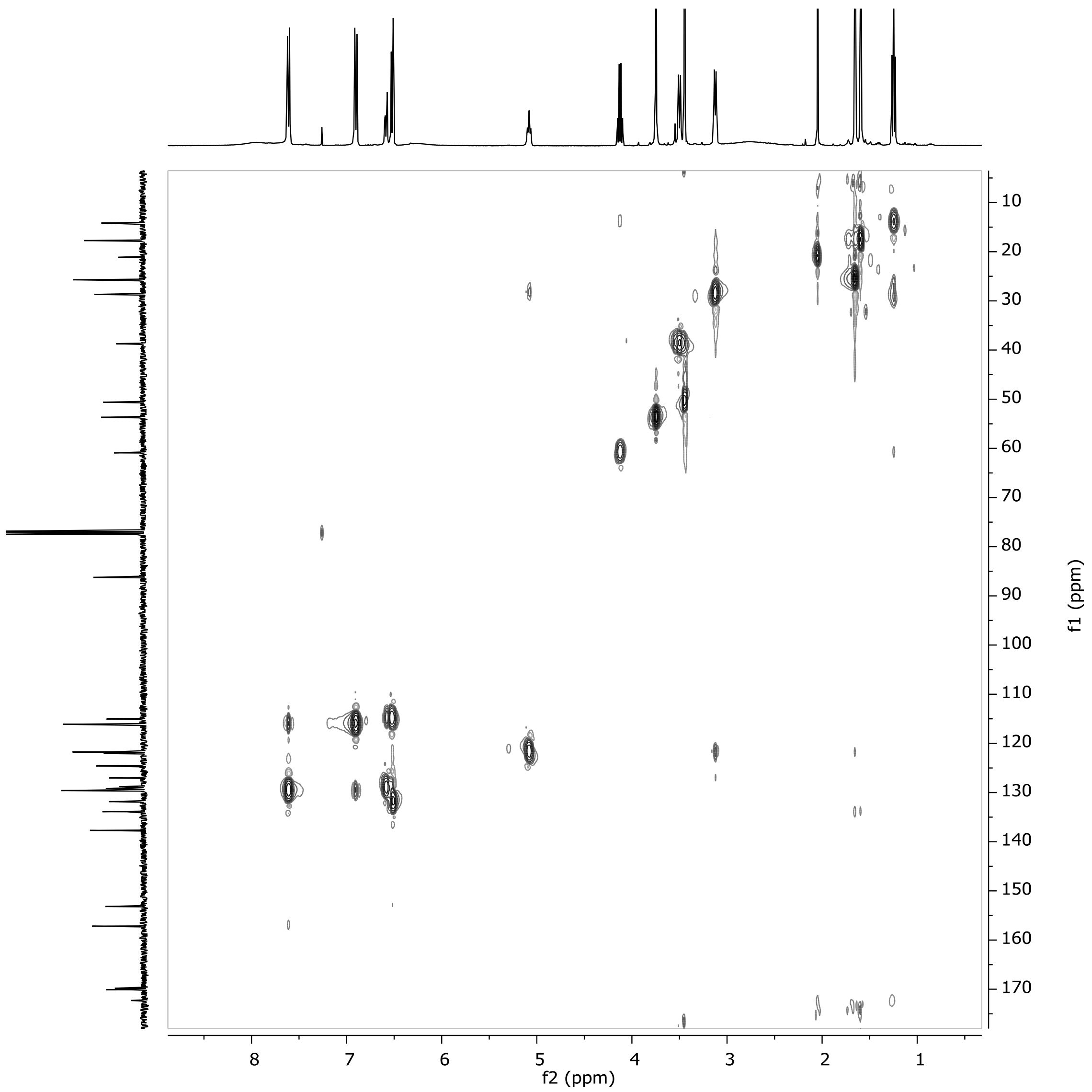

Figure S8. *g*HMQC spectrum of **1** in chloroform-*d*

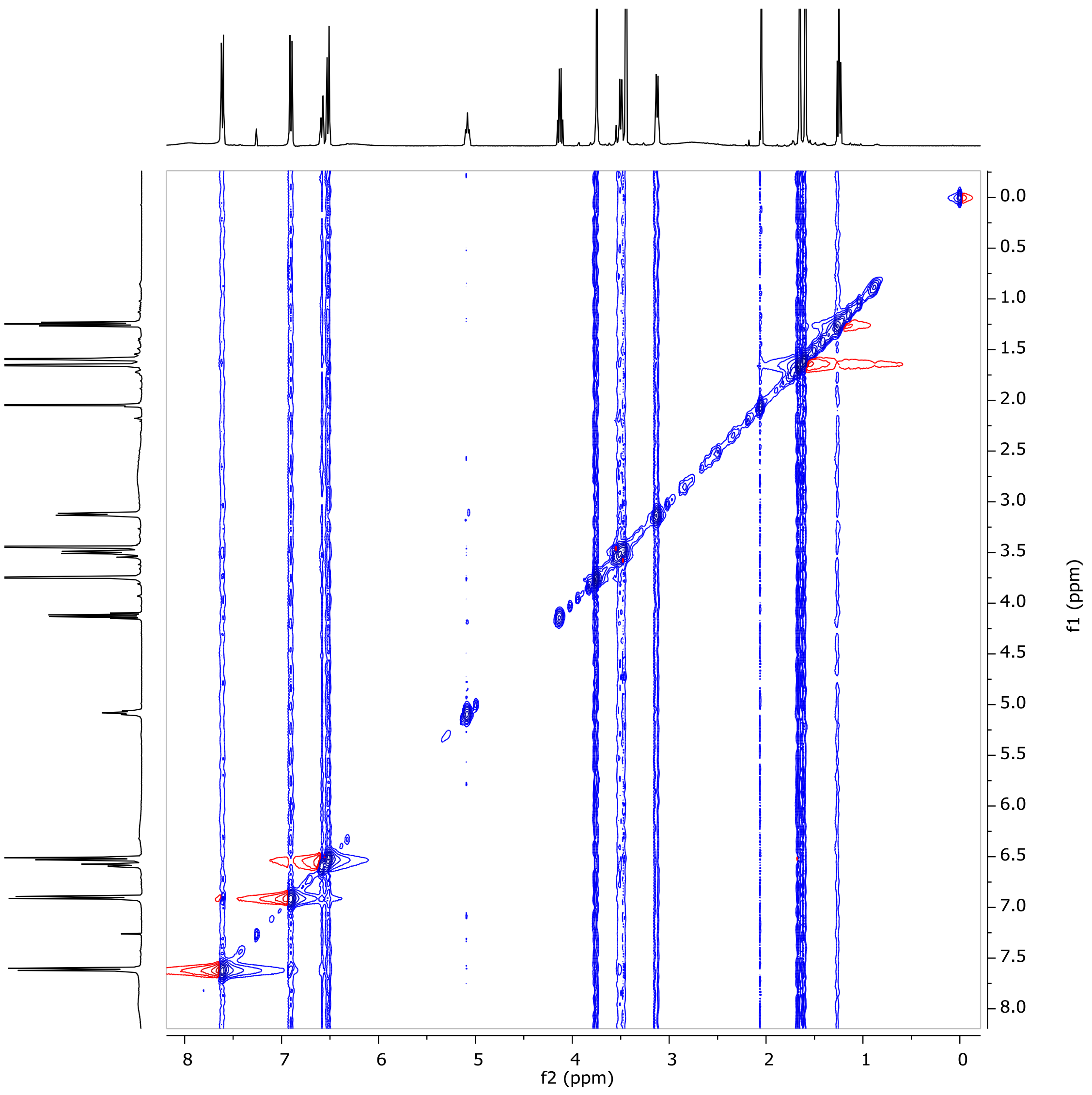

Figure S9. NOESY spectrum of **1** in chloroform-*d*

Table S2. ^{1}H and ^{13}C NMR Data of butyrolactone III (**2**).

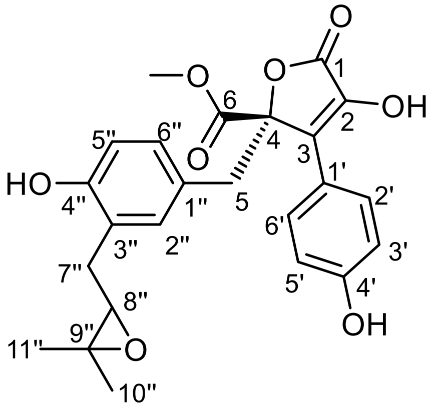

Butyrolactone III (**2**)

Butyrolactone III (**2**) in chloroform-*d*, 600 MHz		
#	δ_H (J[Hz])	δ_C, type
1		169.6, C
2		137.6, C
3		129.7, C
4		86.1, C
5	3.51, d (14.7)<br>3.41, d (14.7)	38.8, CH_2
6		169.9, C
7	3.73, s (3H)	53.7, CH_3
1'		122.0, C
2'	7.56, d (9.0)	129.6, CH
3'	6.90, d (9.0)	116.2, CH
4'		157.3, C
5'	6.90, d (9.0)	116.2, CH
6'	7.56, d (9.0)	129.6, CH
1"		118.4, C
2"	6.51, d (2.1)	132.1, CH
3"		124.8, C
4"		152.0, C
5"	6.53, d (8.1)	116.7, CH
6"	6.51, dd (8.1, 2.1)	128.9, CH
7"	2.82, dd (17.0, 5.0)<br>2.58, dd (17.0, 6.0)	30.9, CH_2
8"	3.74, m	69.7, CH
9"		76.8, C
10"	1.24, s (3H)	22.0, CH_3
11"	1.21, s (3H)	24.8, CH_3

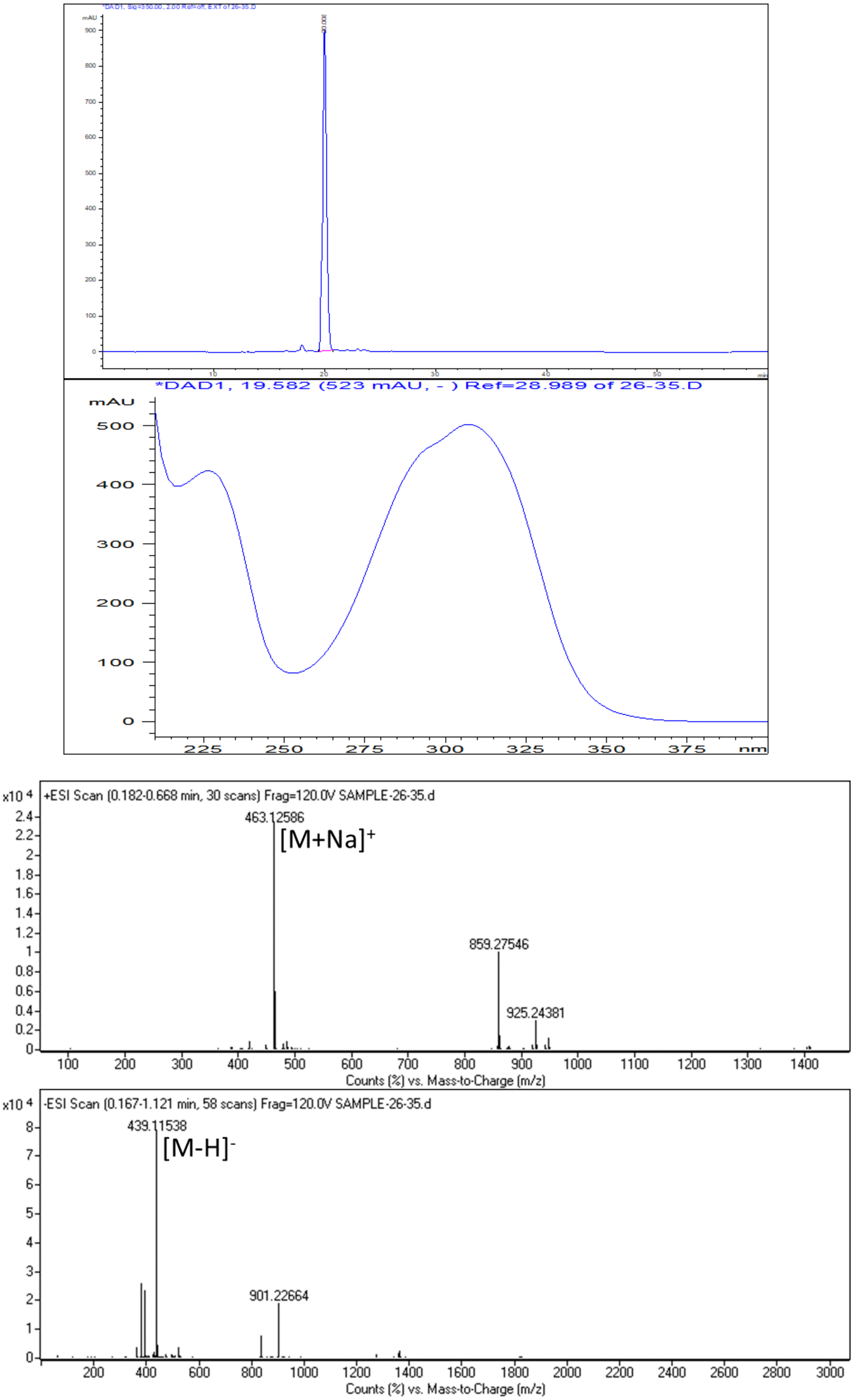

Figure S10. HPLC chromatogram and HRESIMS spectrum of **2**

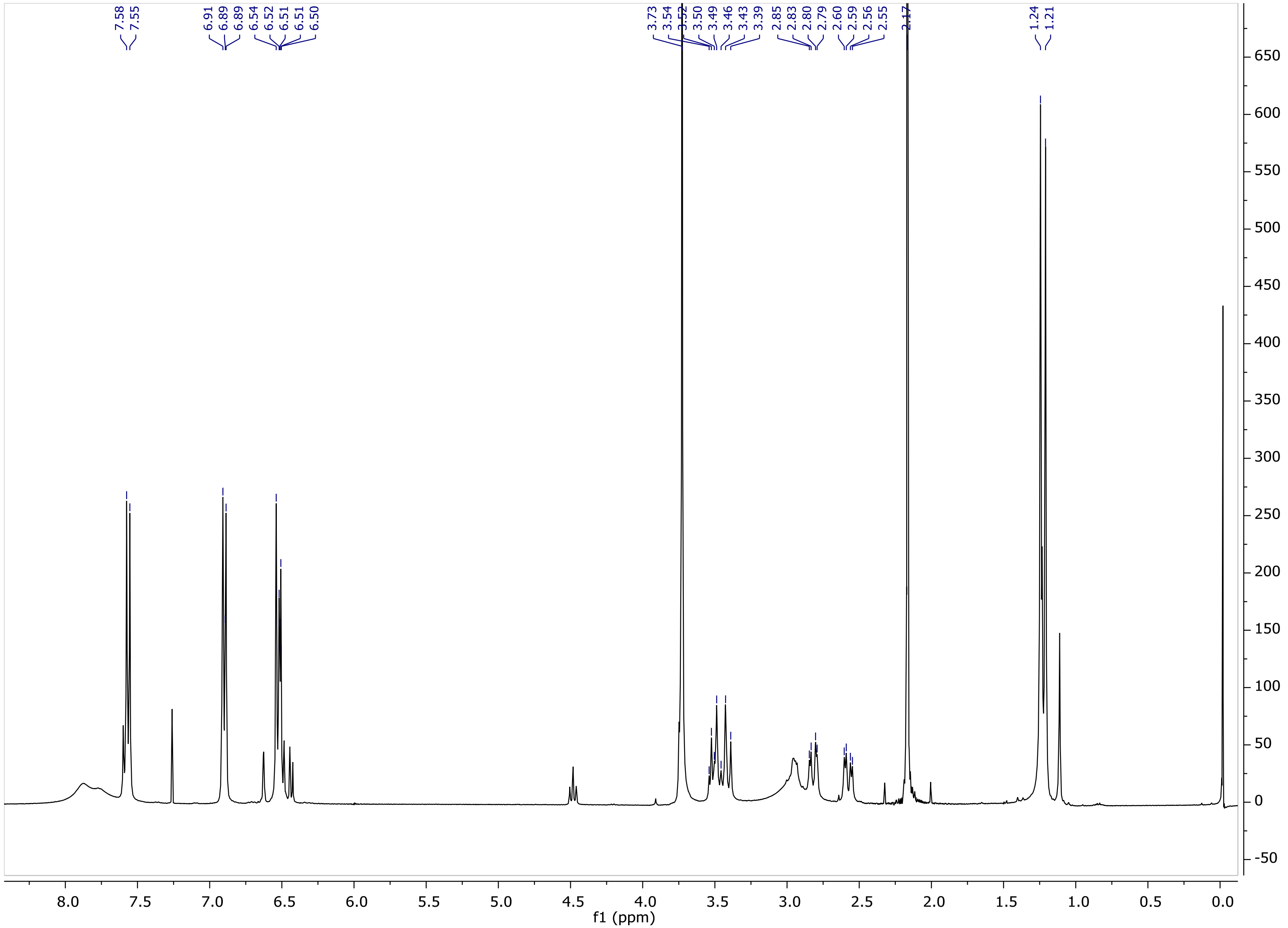

Figure S11. ^{1}H-NMR spectrum of **2** in chloroform-*d*

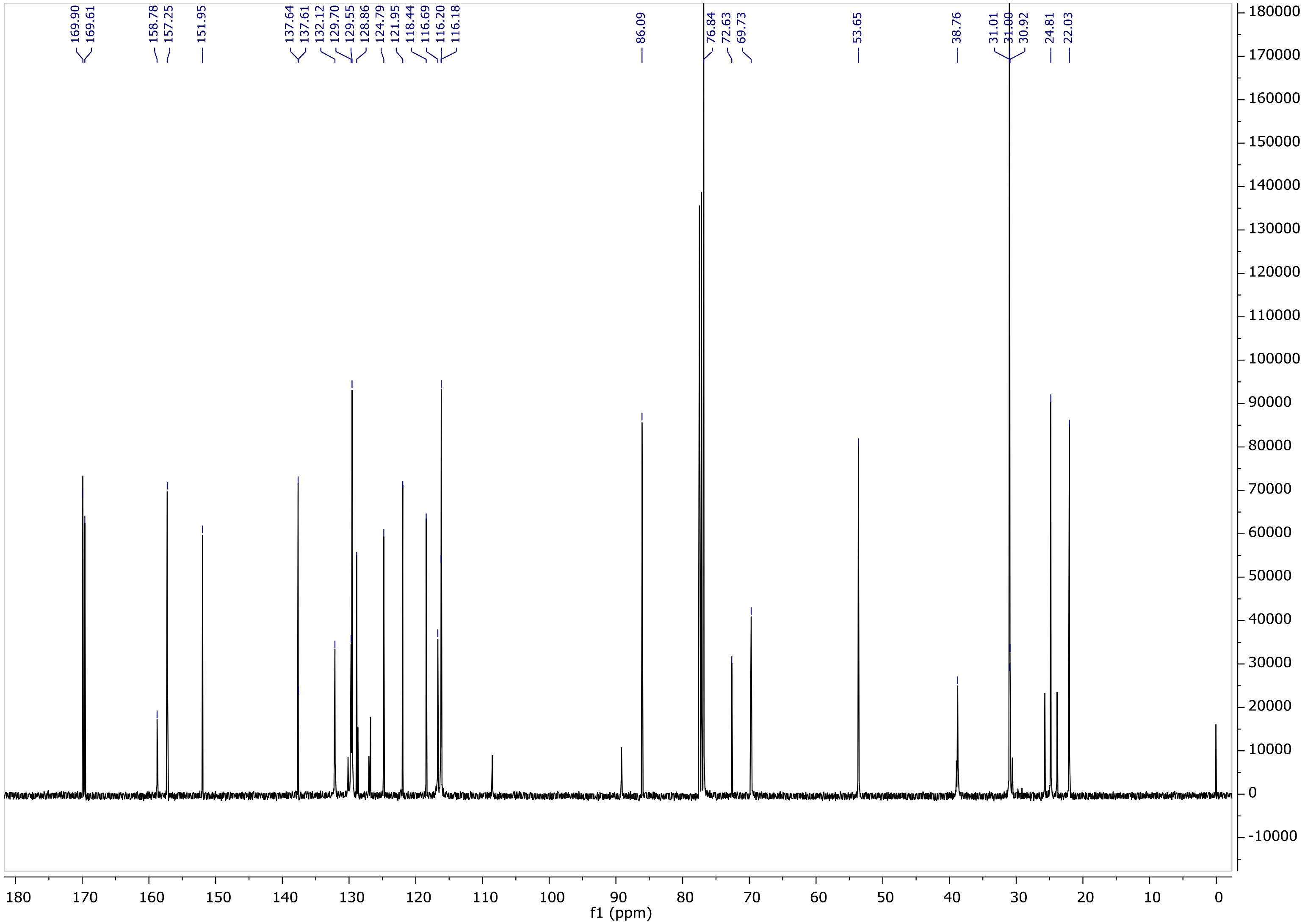

Figure S12. ^{13}C-NMR spectrum of **2** in chloroform-*d*

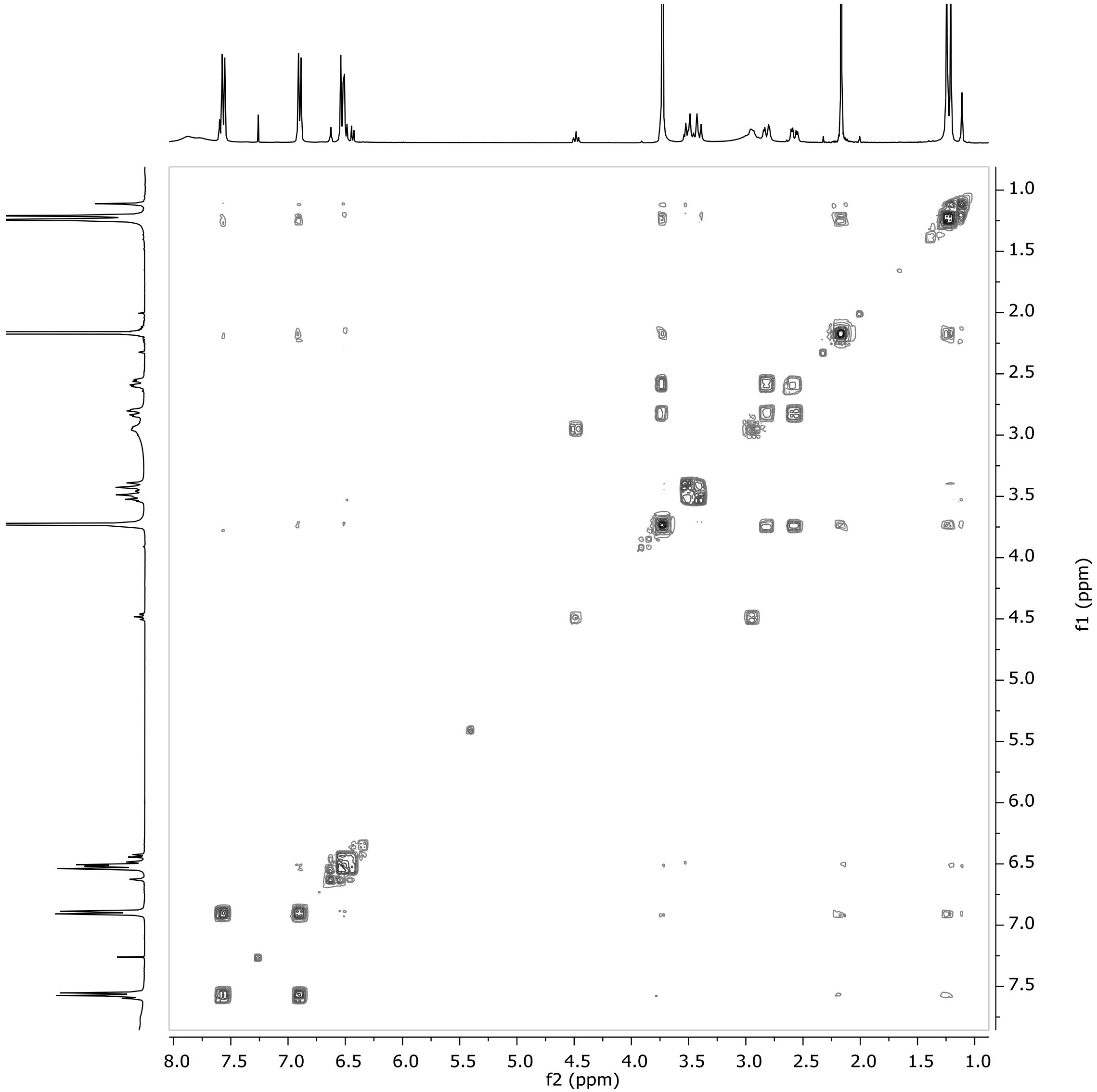

Figure S13. $^1H–^1H$ COSY spectrum of **2** in chloroform-*d*

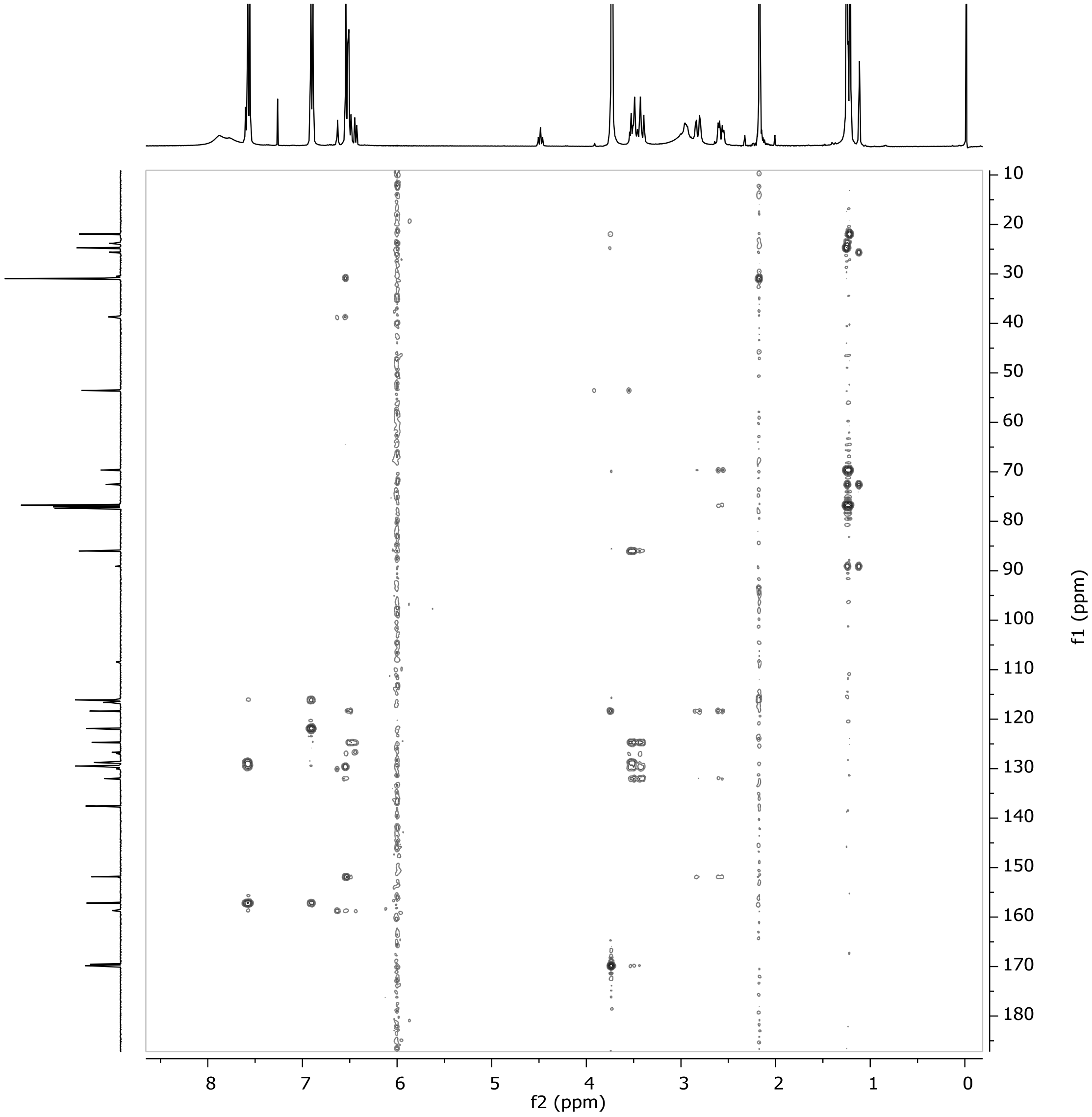

Figure S14. *g*HMBC spectrum of **2** in chloroform-*d*

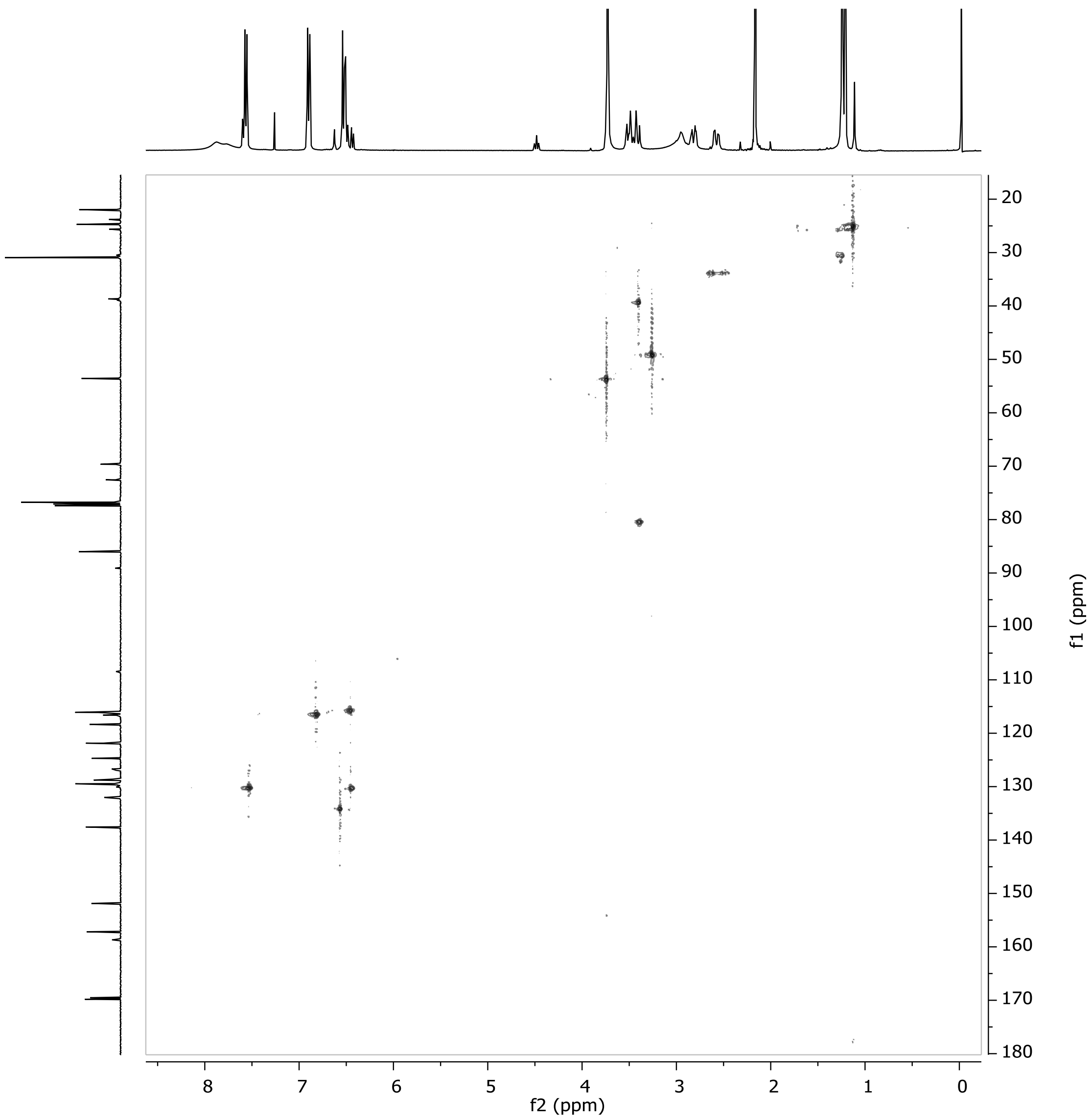

Figure S15. *g*HMQC spectrum of **2** in chloroform-*d*

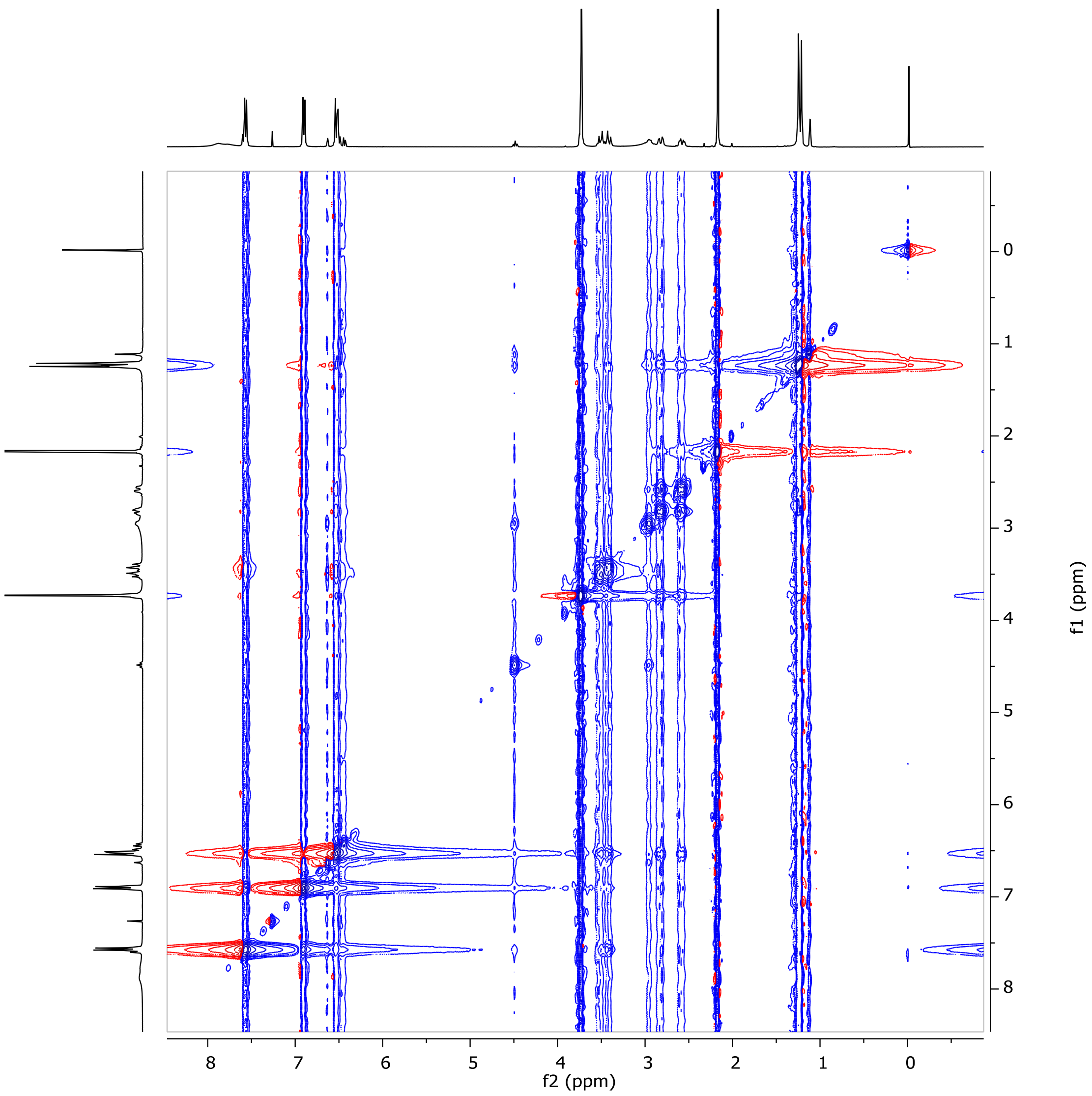

Figure S16. NOESY spectrum of **2** in chloroform-*d*

Table S3. ^{1}H and ^{13}C NMR Data of terretonin (**3**).

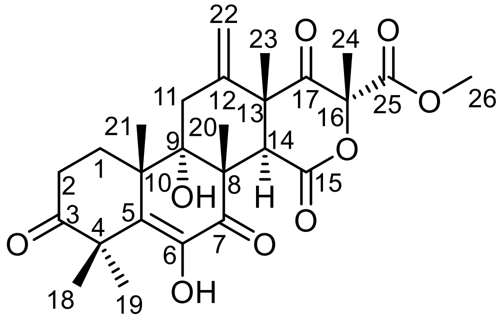

Terretonin (**3**)

Terretonin (**3**) in chloroform-*d*, 400 MHz		
#	δ_H (*J*[Hz])	δ_C, type
1	2.36, dd (13.1, 9.3)	28.3, CH_2
	1.76, dd (13.1, 8.3)	
2	2.70, dd (19.0, 8.6)	32.8, CH_2
	2.52, ddd (19.2, 11.3, 8.5)	
3		214.2, CO
4		47.9, C
5		131.7, C
6		138.8, C
7		197.1, CO
8		52.5, C
9		77.7, C
10		43.3, C
11	2.96, dt (14.2, 1.6)	35.0, CH_2
	2.27, d (14.2)	
12		139.9, C
13		49.6, C
14	3.54, s	44.7, CH
15		167.9, CO
16		85.6, C
17		201.6, CO
18	1.46, s (3H)	23.7, CH_3
19	1.46, s (3H)	23.7, CH_3
20	1.94, s (3H)	19.9, CH_3
21	1.21, s (3H)	18.7, CH_3
22	5.08, d (1.5)	117.2, CH_2
	5.45, d (1.5)	
23	1.43, s (3H)	23.5, CH_3
24	1.72, s (3H)	21.4, CH_3
25		168.7, CO
26	3.79, s (3H)	53.8, CH_3
6-OH	6.14, s	

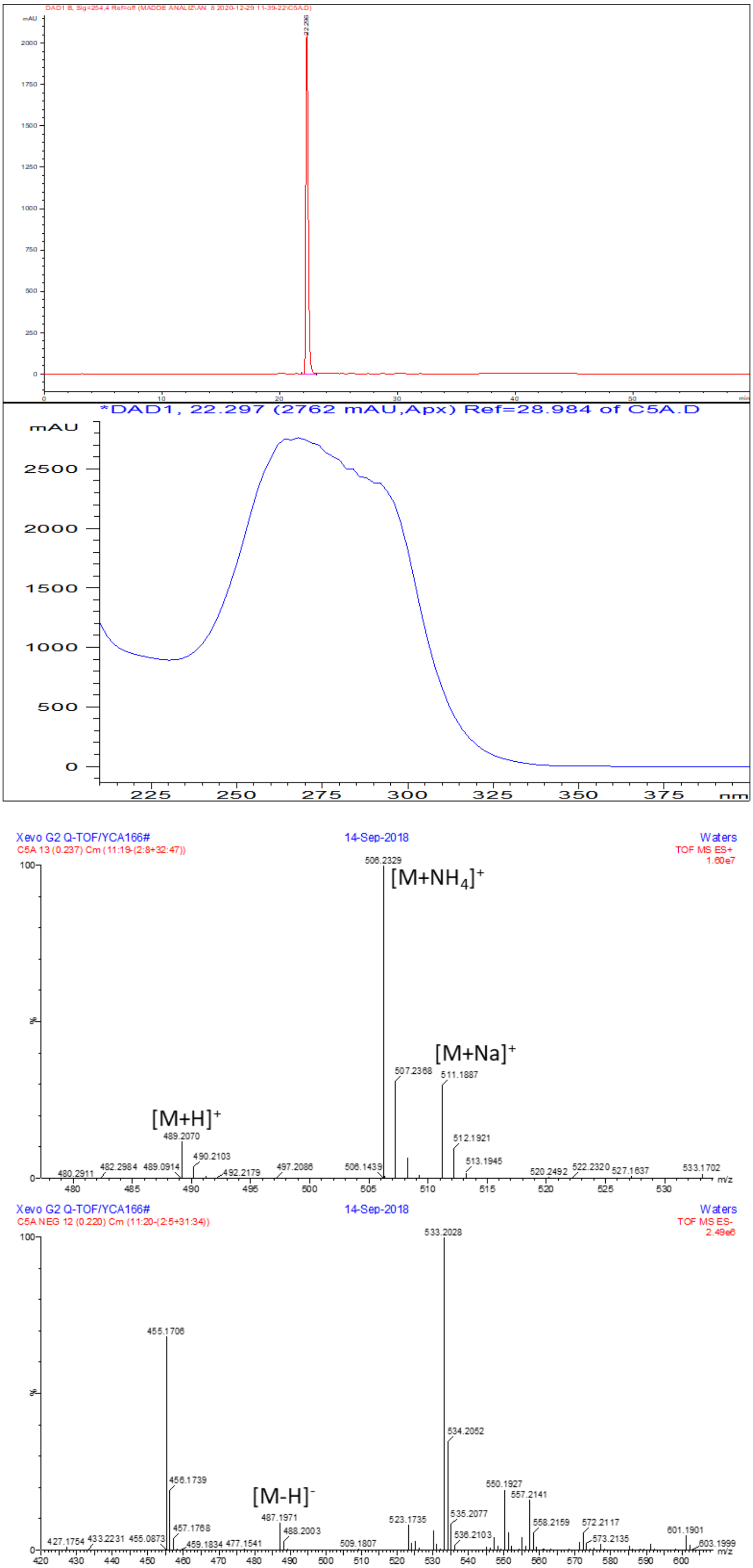

Figure S17. HPLC chromatogram and HRESIMS spectrum of **3**

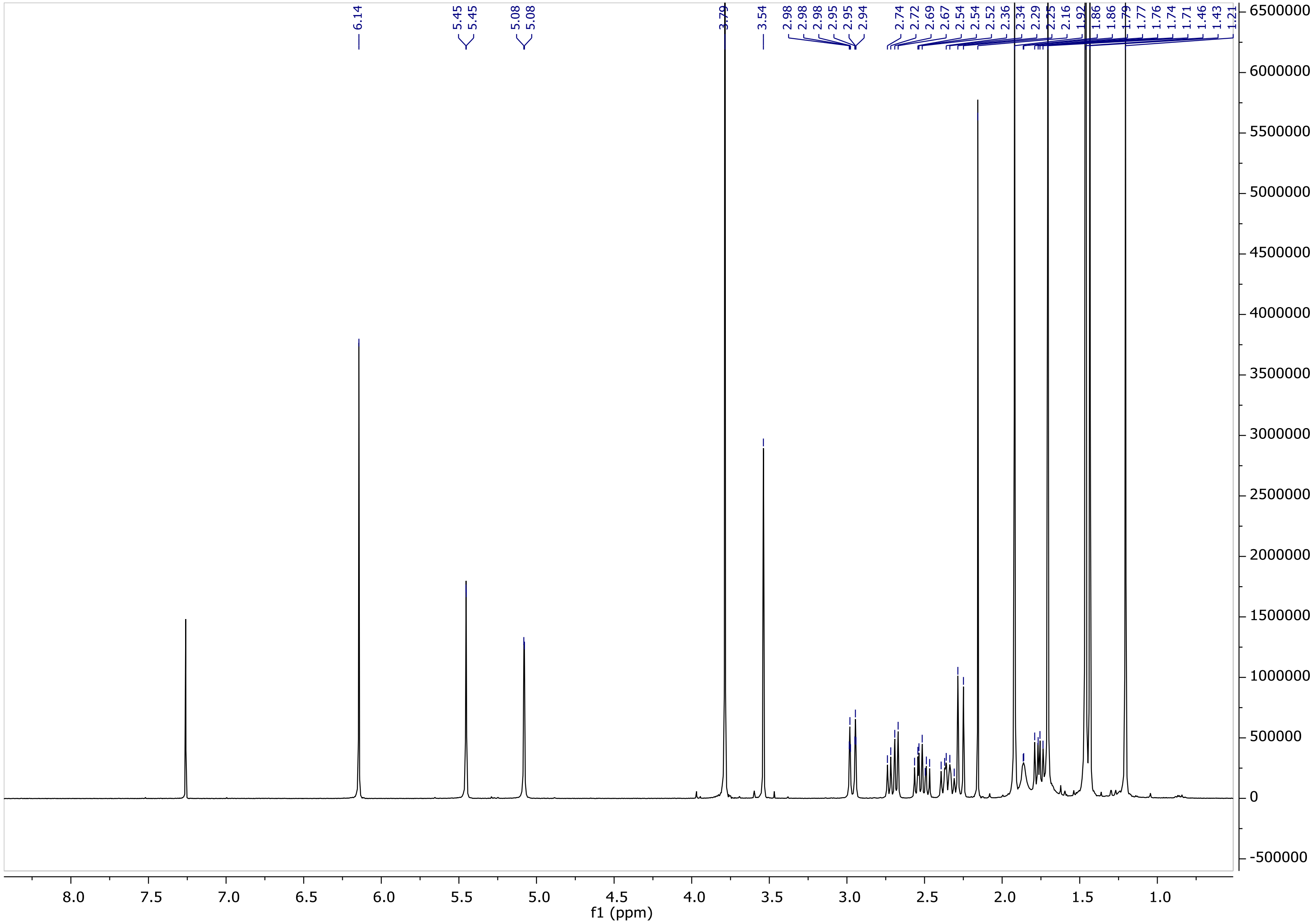

Figure S18. ^{1}H-NMR spectrum of **3** in chloroform-*d*

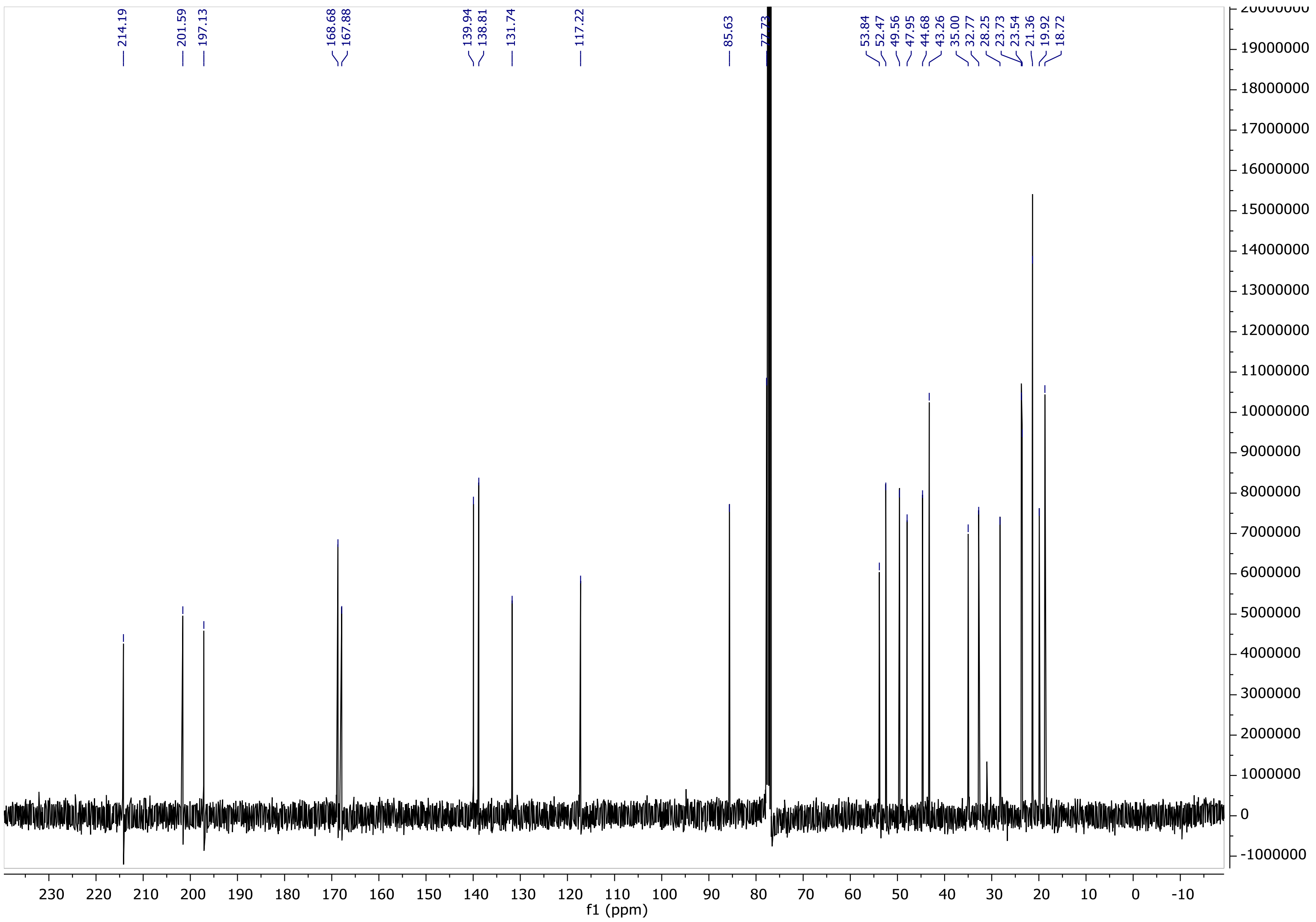

Figure S19. ^{13}C-NMR spectrum of **3** in chloroform-*d*

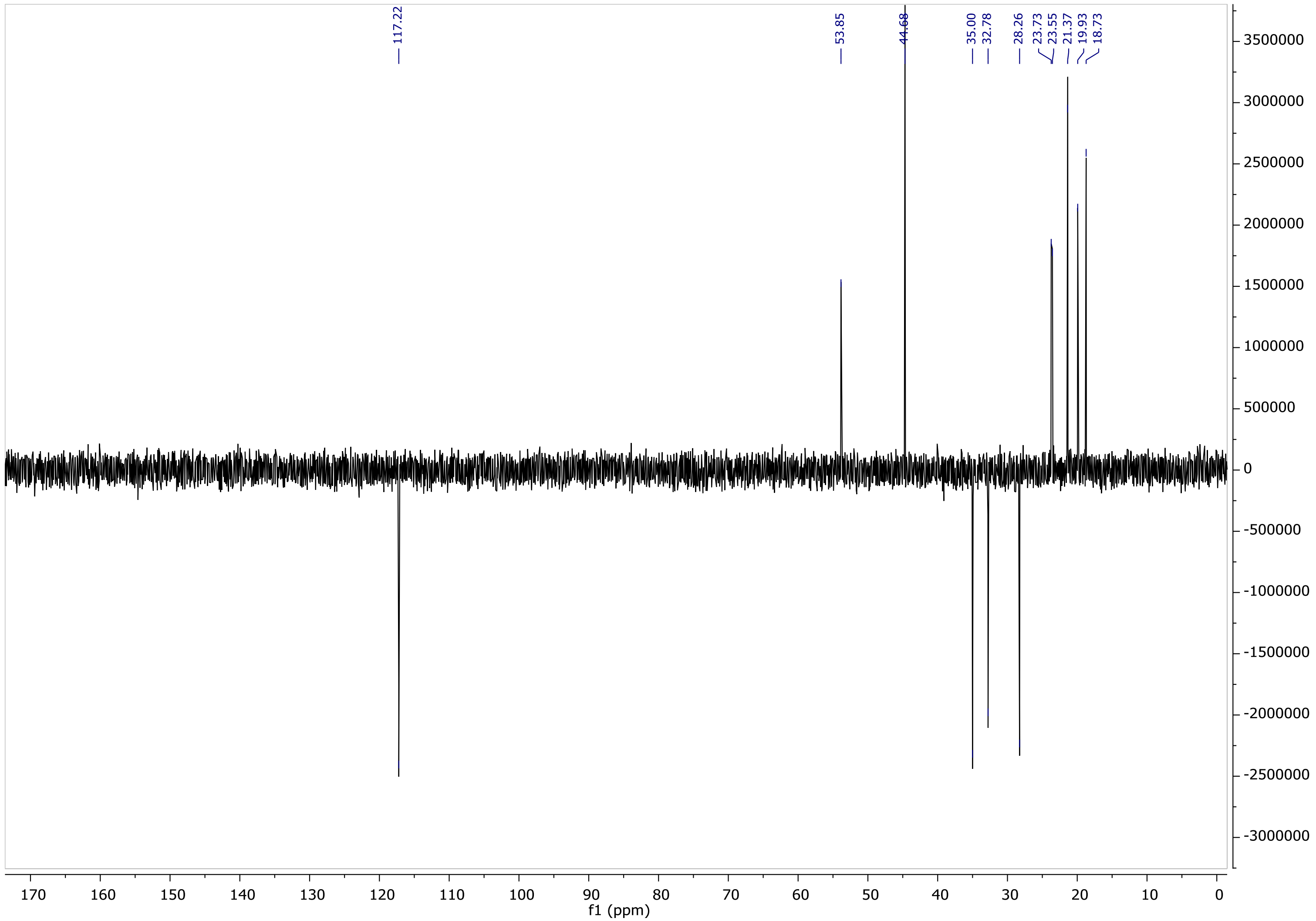

Figure S20. DEPT spectrum of **3** in chloroform-*d*

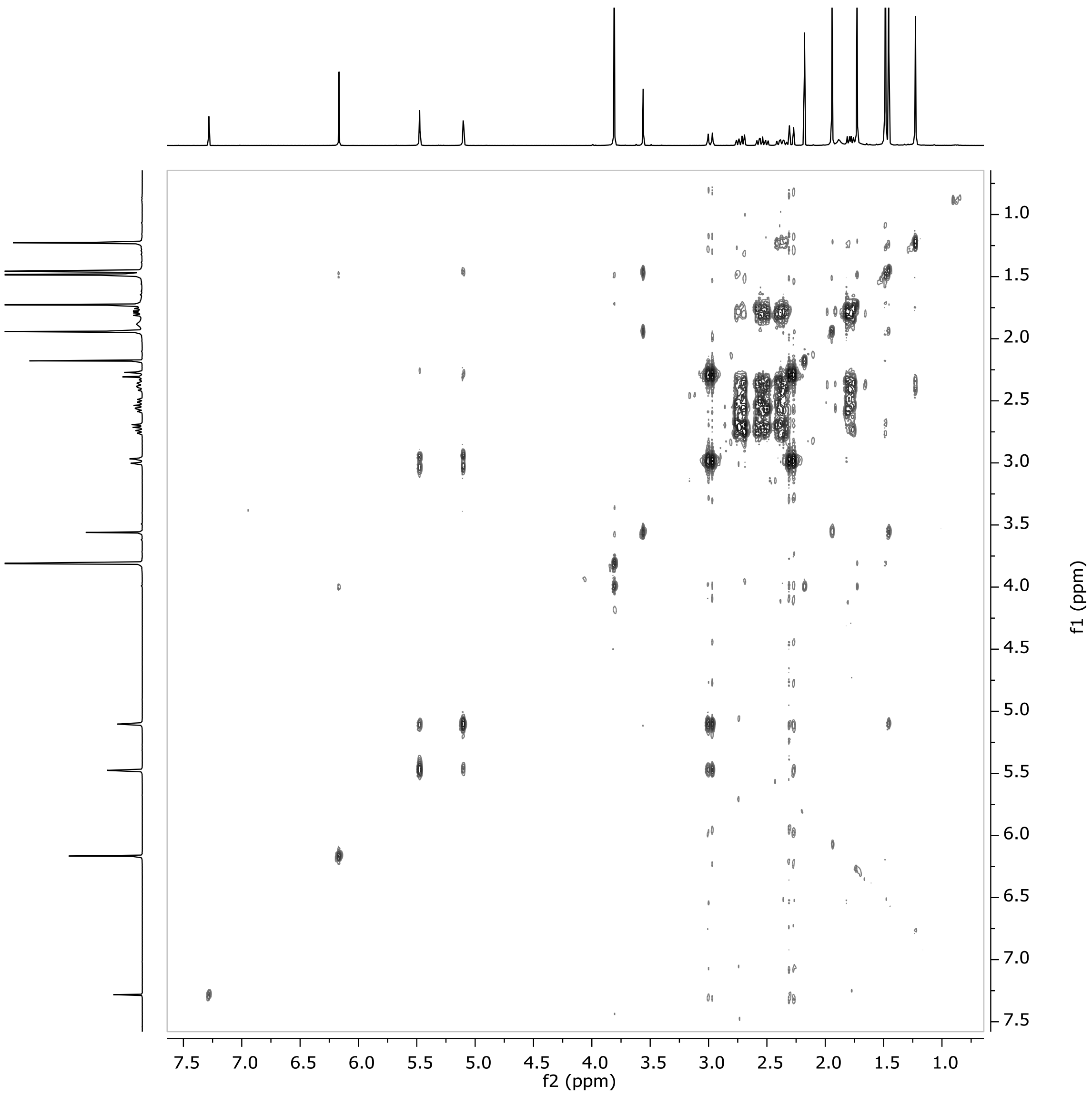

Figure S21. ^{1}H–^{1}H COSY spectrum of **3** in chloroform-*d*

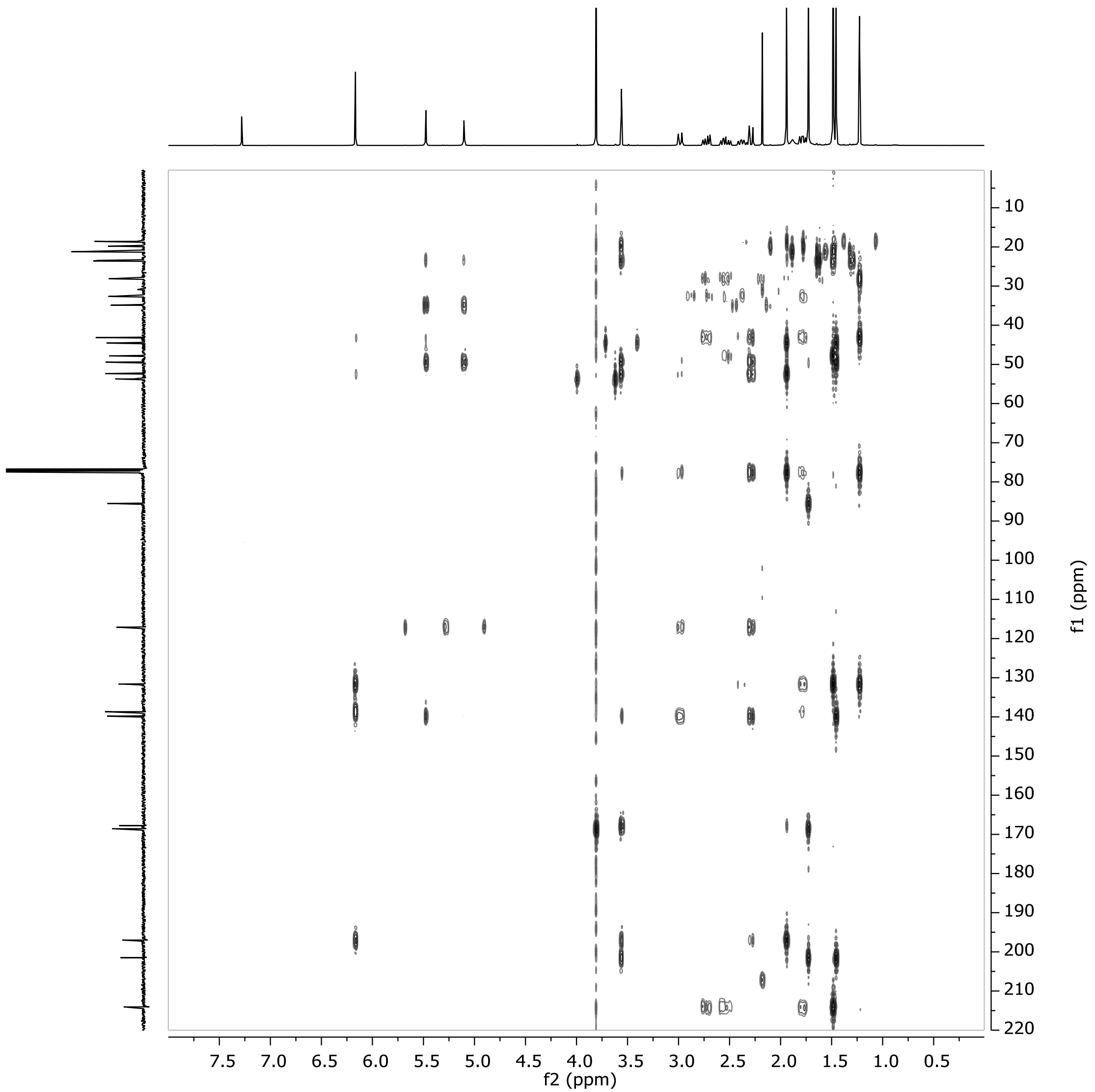

Figure S22. *g*HMBC spectrum of **3** in chloroform-*d*

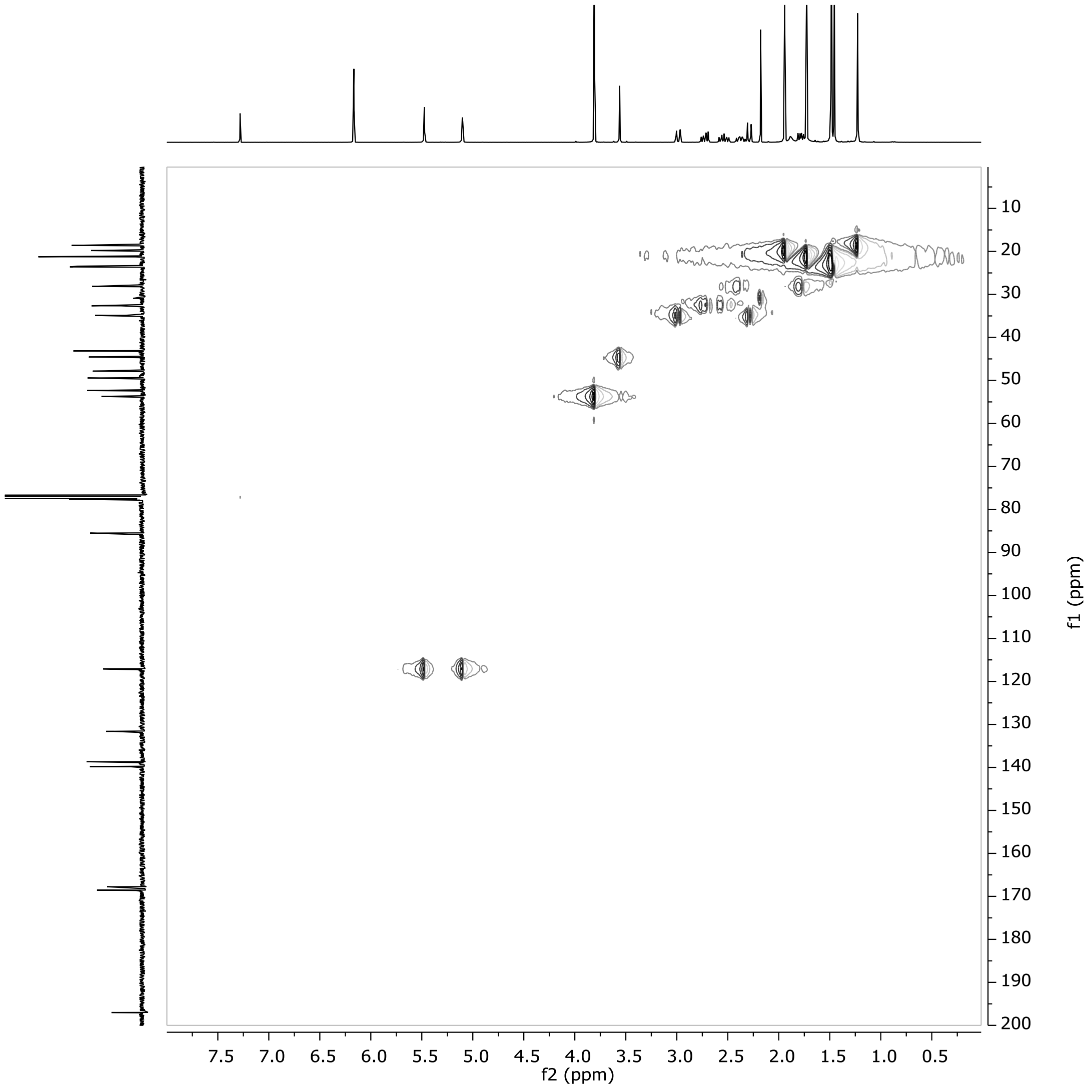

Figure S23. *g*HMQC spectrum of **3** in chloroform-*d*

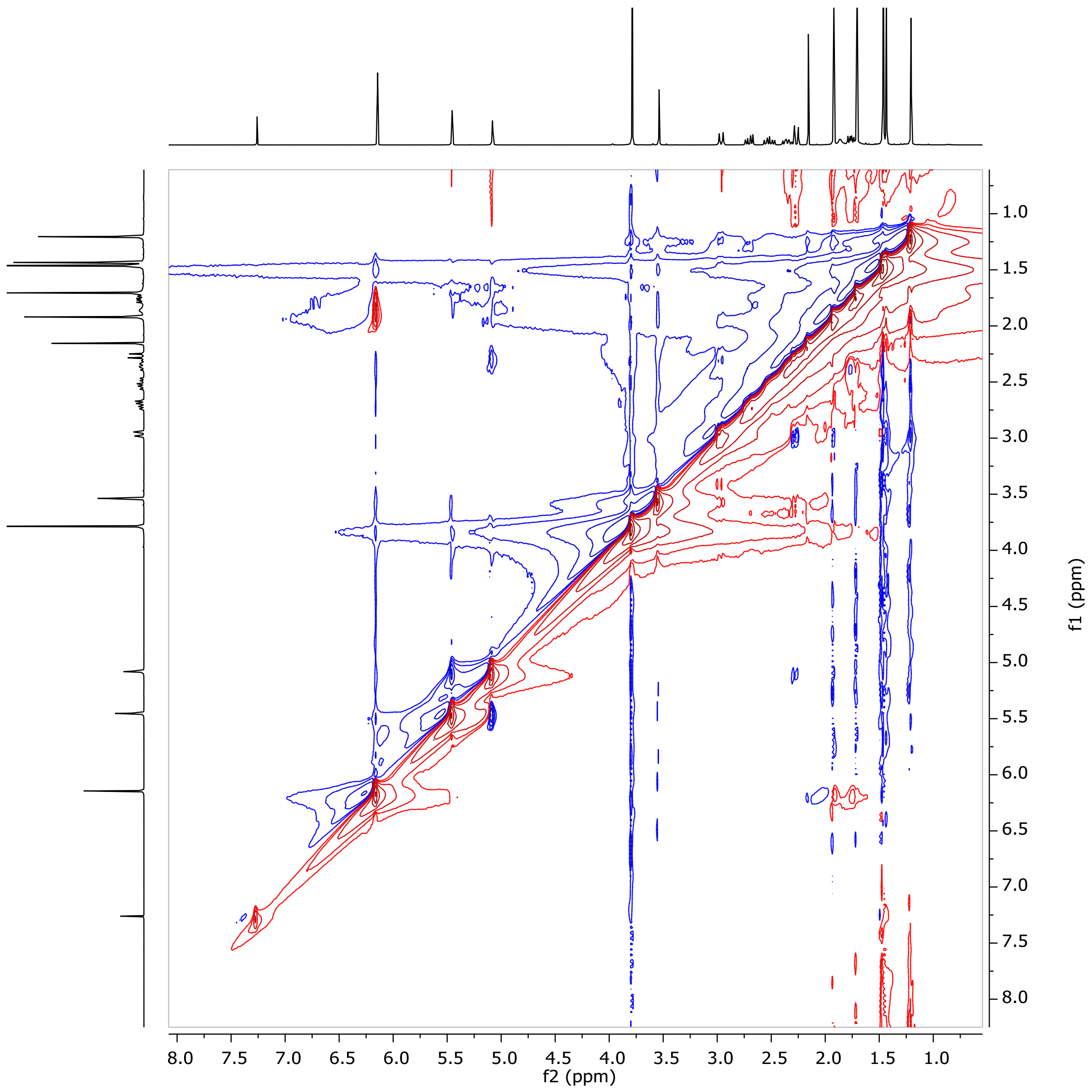

Figure S24. NOESY spectrum of **3** in chloroform-*d*

Table S4. ^{1}H and ^{13}C NMR Data of 4-hydroxy-3-(3-methylbut-2-enyl)benzaldehyde (**4**).

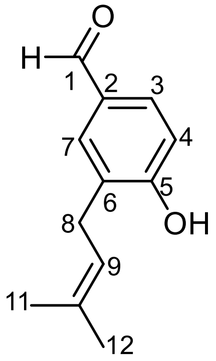

4-Hydroxy-3-(3-methylbut-2-enyl)benzaldehyde (**4**)

4-Hydroxy-3-(3-methylbut-2-enyl)benzaldehyde (**4**) in DMSO-d_6, 600 MHz					
#	δ_H (J[Hz])	δ_C, type	#	δ_H (J[Hz])	δ_C, type
1	9.75, s	191.1, CO	7	7.58, d (2.2)	130.7, CH
2		128.4, C	8	3.26, d (7.4, 2H)	27.7, CH_2
3	7.60, dd (8.2, 2.2)	130.1, CH	9	5.29, td (7.4,1.5)	121.9, CH
4	6.95, d (8.2)	115.1, CH	10		132.2, C
5		161.1, CO	11	1.71, d (1.5, 3H)	25.5, CH_3
6		128.5, C	12	1.68, d (1.5, 3H)	17.6, CH_3
5-OH	10.58, s				

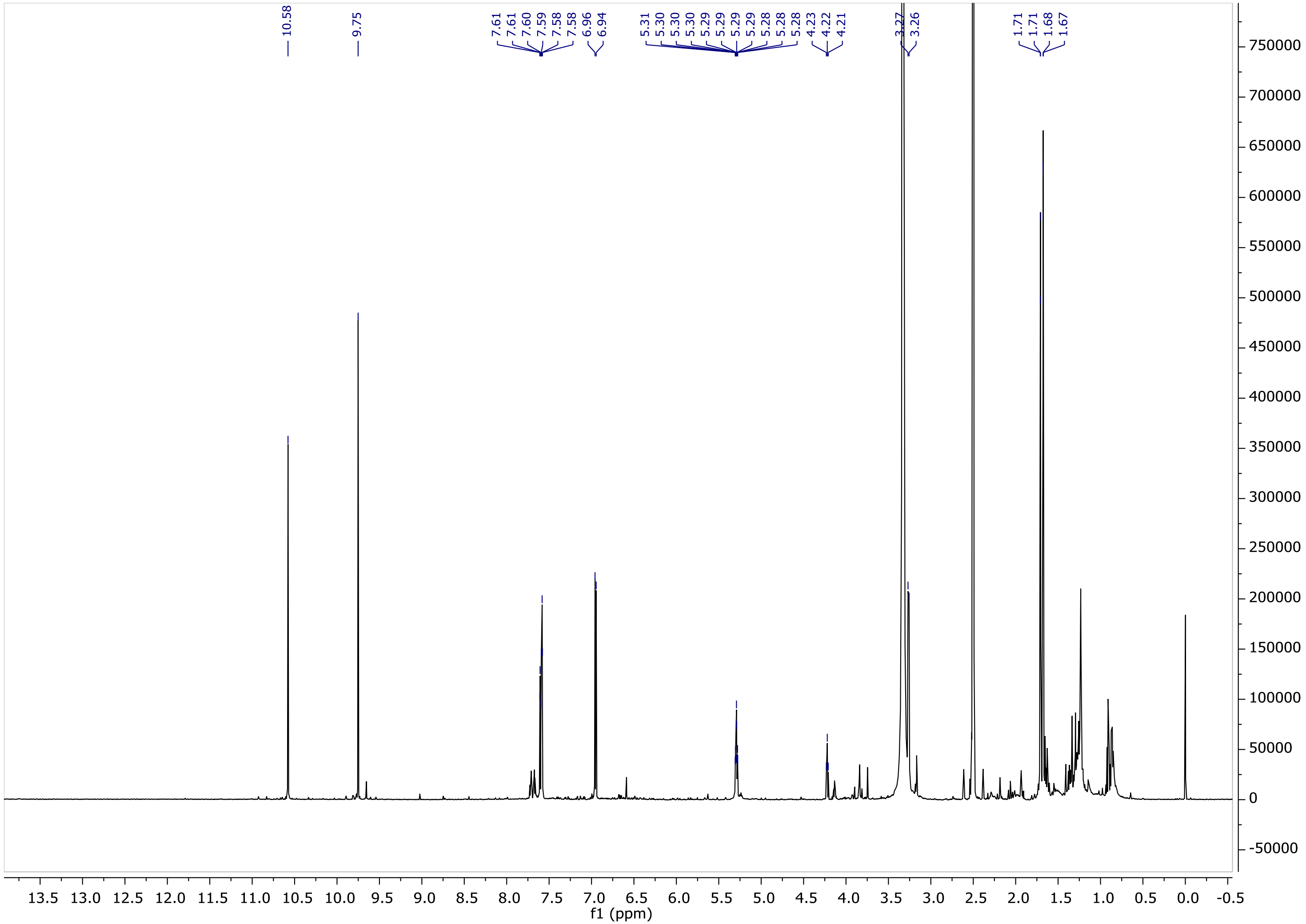

Figure S25. ^{1}H-NMR spectrum of **4** in DMSO-d_6

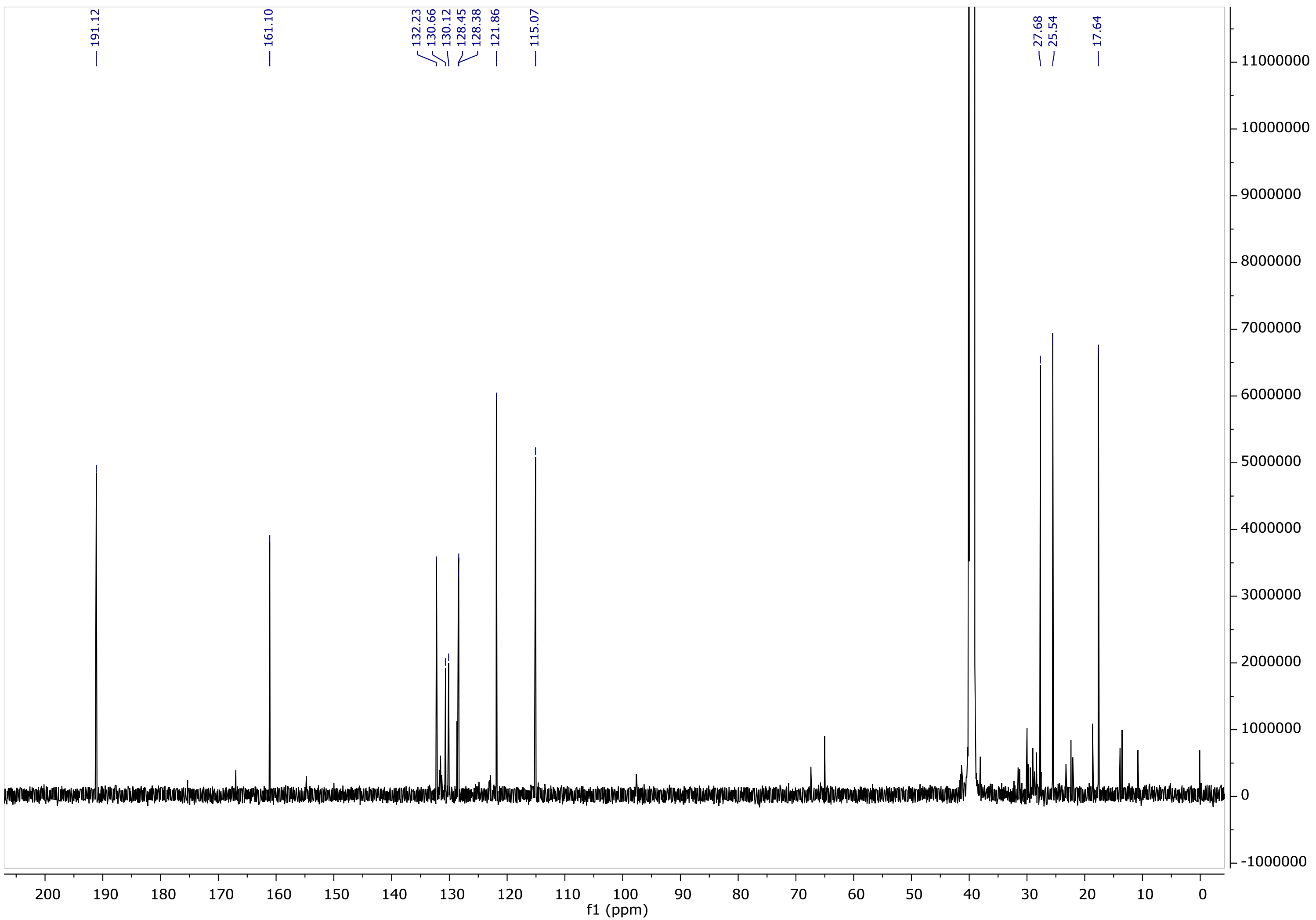

Figure S26. ^{13}C-NMR spectrum of **4** in DMSO-d_6

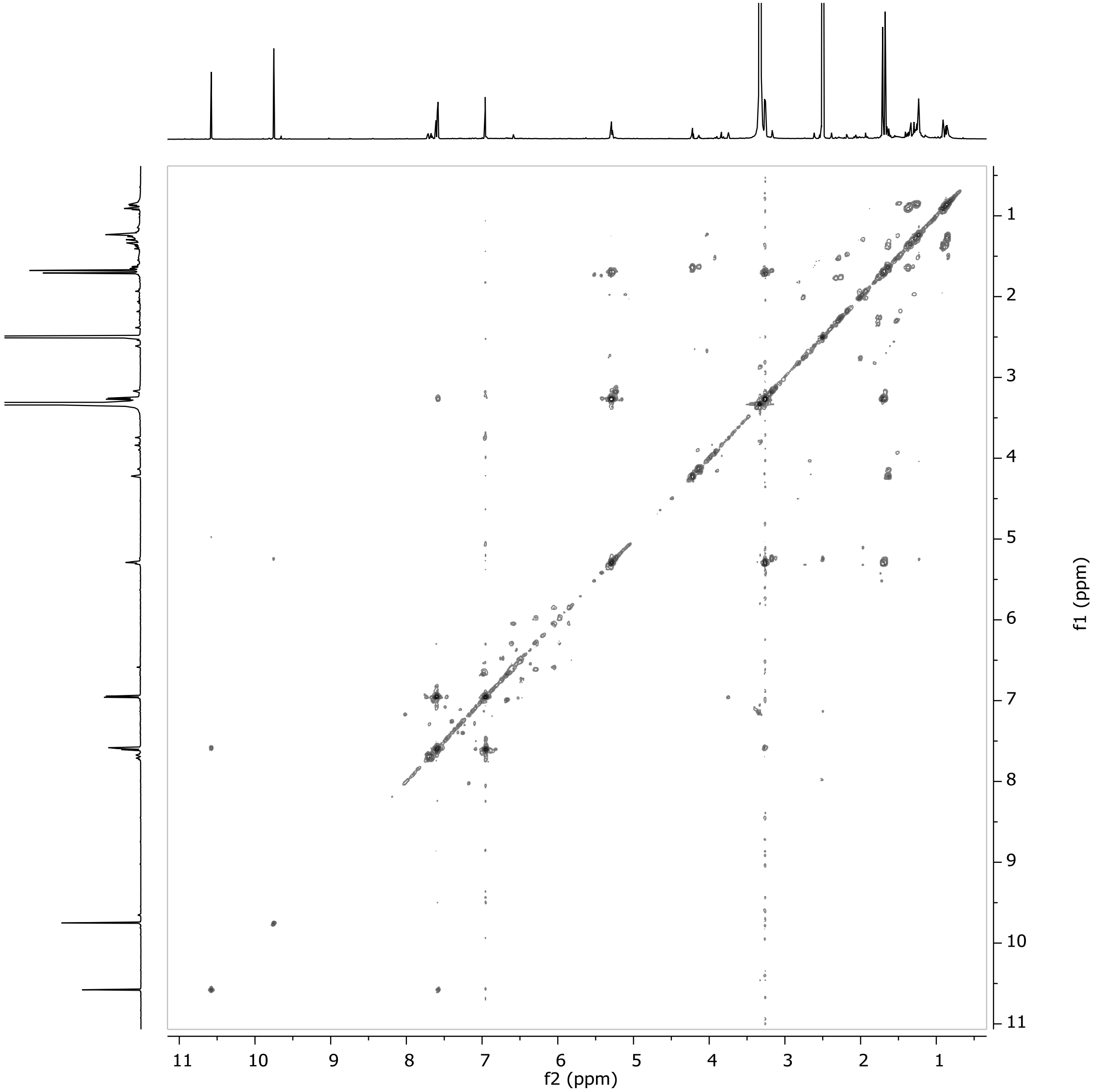

Figure S27. ^1H–^1H COSY spectrum of **4** in DMSO-d_6

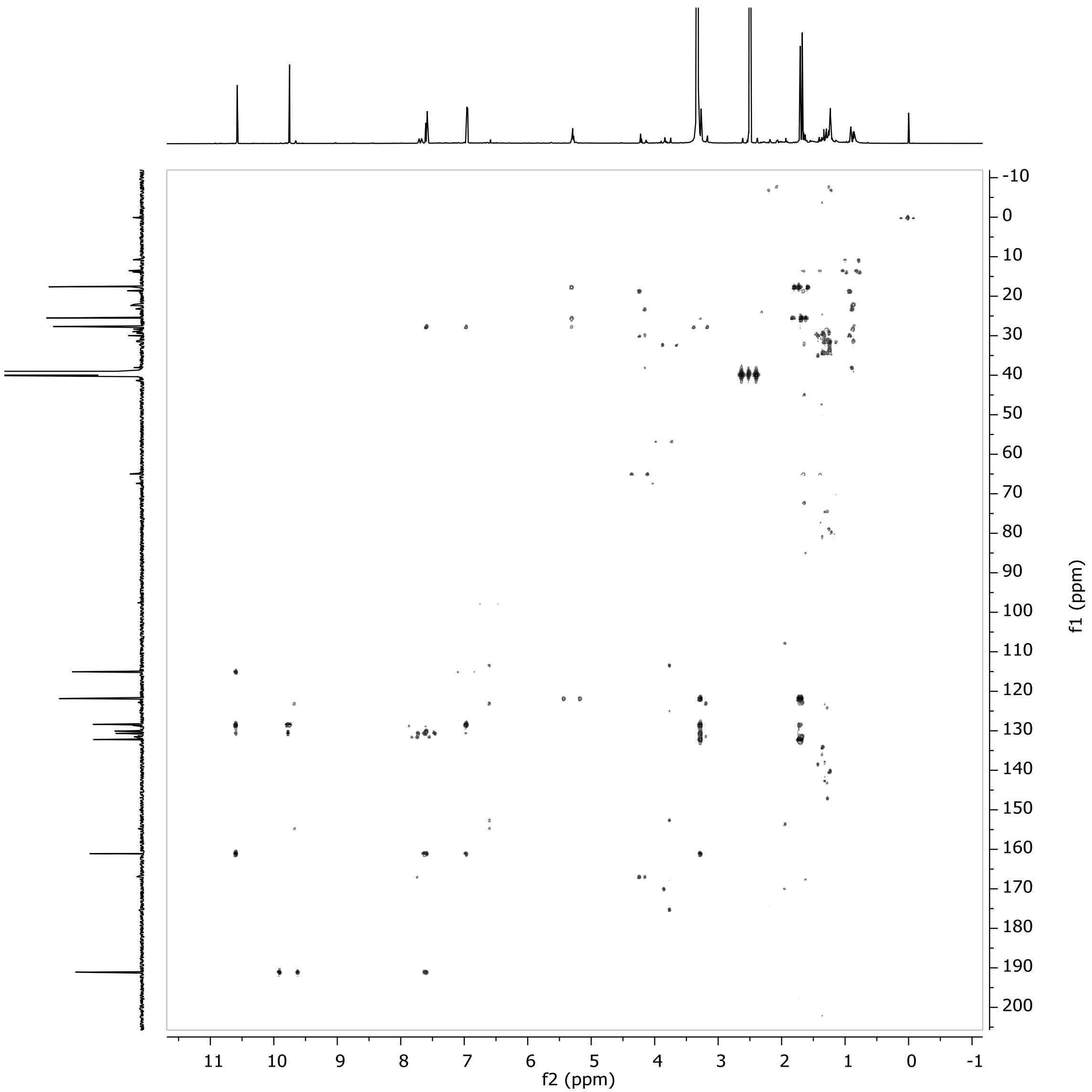

Figure S28. *g*HMBC spectrum of **4** in DMSO-d_6

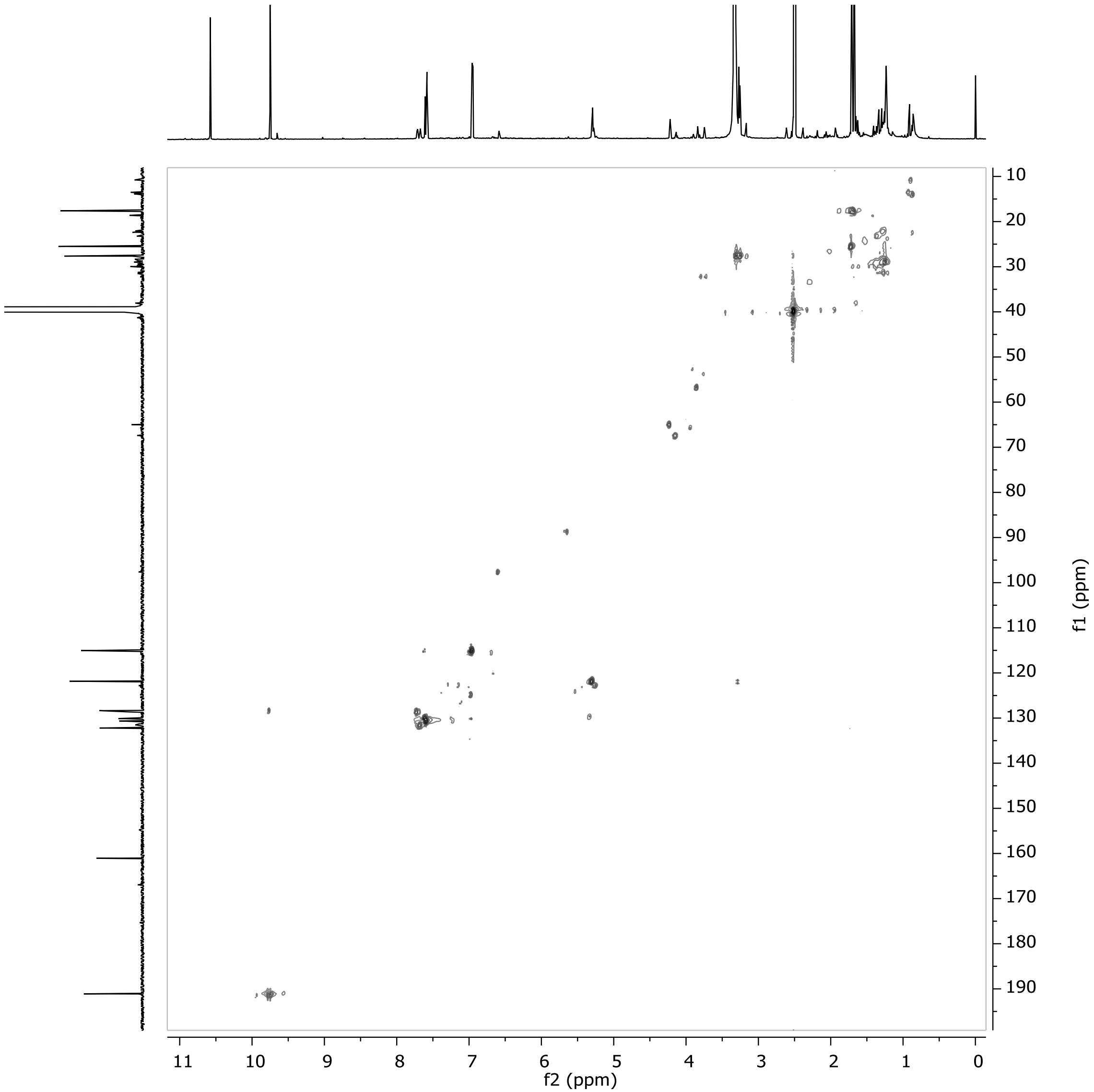

Figure S29. *g*HMQC spectrum of **4** in DMSO-d_6
